# Main Group Multiple Bonds for Bond Activations and Catalysis

**DOI:** 10.1002/chem.202002939

**Published:** 2020-11-19

**Authors:** Catherine Weetman

**Affiliations:** ^1^ WestCHEM Department of Pure and Applied Chemistry University of Strathclyde Glasgow G1 1XL UK

**Keywords:** bond activation, catalysis, main group, multiple bonds, small molecule activation

## Abstract

Since the discovery that the so‐called “double‐bond” rule could be broken, the field of molecular main group multiple bonds has expanded rapidly. With the majority of homodiatomic double and triple bonds realised within the *p*‐block, along with many heterodiatomic combinations, this Minireview examines the reactivity of these compounds with a particular emphasis on small molecule activation. Furthermore, whilst their ability to act as transition metal mimics has been explored, their catalytic behaviour is somewhat limited. This Minireview aims to highlight the potential of these complexes towards catalytic application and their role as synthons in further functionalisations making them a versatile tool for the modern synthetic chemist.

## Graphical abstract

**The versatile reactivity** of molecular main group multiple bonds is covered in this Minireview, from small molecule activation to catalysis and to their role as synthons in further functionalisation reactions.

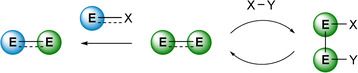

## Introduction

Molecular main group multiple bond chemistry has rapidly developed since the isolation of the first silicon‐silicon double bond. West's disilene^[1]^ broke the so called “double‐bond” rule, in which it was thought that p‐block elements with a principal quantum number greater than two (i.e. aluminium onwards) would not form multiple bonds with themselves or other elements. Seminal examples, all from 1981, reported by West (Si=Si), Yoshifuji (P=P),^[2]^ Brook (Si=C),^[3]^ and Becker (P≡C)^[4]^ paved the way for this new field. Almost 40 years on, homodiatomic double bonds have now been isolated for all *p*‐block elements in groups 13–15, rows 2–6.[^5^, ^6^, ^7^] Extension to homodiatomic triple bonds is complete for group 14, whilst only one clear example of a group 13 triple bond exists.^[8]^ Further advances in heterodiatomic multiple bonding for p‐block elements has yielded many new complexes, yet several still remain elusive.

**Figure 1:**
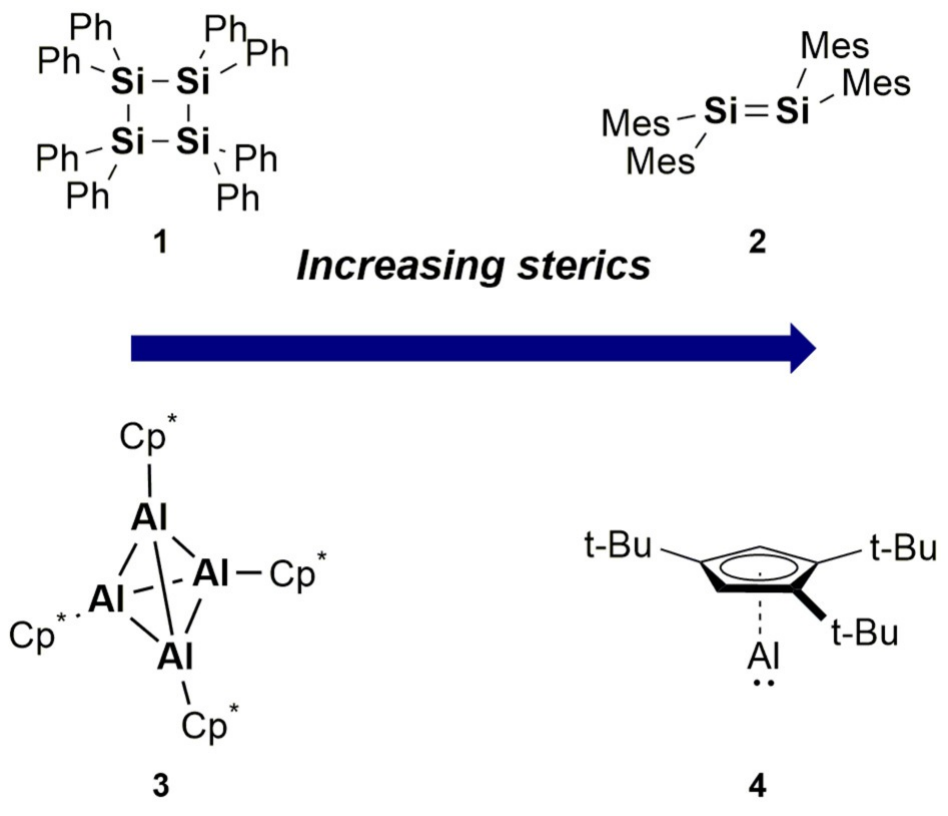
Effect of ligand steric demands on isolable structures. Mes=2,4,6‐trimethylphenyl, Cp*=1,2,3,4,5‐pentamethylcyclopentadienyl.

Synthetic challenges in main group multiple bond chemistry have largely been overcome through choice of supporting ligand. Careful design of sterically demanding ligands is required in order to provide sufficient kinetic stabilisation to the multiple bond (Figure 1). If too small higher oligomers are obtained or if too large steric clash will prevent multiple bond formation. For example, on increasing the steric demands of phenyl to mesityl (mesityl=2,4,6‐trimethylphenyl) multiple bond formation is achieved (Compounds **1** ^[9]^ vs. **2**,^[1]^ Figure 1). Similarly, the widely studied tetrameric pentamethylcyclopentadiene (Cp*) aluminium complex (**3**)^[10]^ dissociates into its monomeric form at elevated temperatures. It was only recently, however, that a monomeric Cp‐derived Al^I^ species was isolated (**4**)^[11]^ through increasing the steric demands of the substituents. It is of note, that no dimeric (i.e. multiple bond) structure has been observed for the aluminium Cp systems.

On descending the group the stability of the lower oxidation state increases and thus its desire to partake in bond formation decreases. For example in group 14, Sn^II^ is more stable than Sn^IV^, whilst for the lightest congener C^IV^ is more stable than C^II^. This can also influence the complex formation in both the solution and solid state as highlighted by Lappert's stannylene. The use of a bis(trimethylsilyl)methyl ligand (CH(SiMe_3_)_2_) provides sufficient kinetic stabilisation to isolate a two‐coordinate Sn^II^ compound, however in the solid state this exists as a dimer yielding a Sn=Sn multiple bond.[^12^, ^13^]

**Figure 2:**
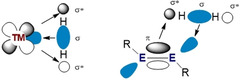
Frontier molecular orbitals of transition metals (left) and main group multiple bonds (right) for the activation of dihydrogen.

One of the reasons for the rapid development of main group multiple bonds is due to their ability to act as transition metal mimics. Owing to similarly energetically accessible frontier orbitals, main group multiple bonds have been shown to activate small molecules, such as dihydrogen, under ambient conditions (Figure 2).[^14^, ^15^] This often results in an oxidative addition reaction occurring at the main group centre. However, unlike transition metals, reductive elimination at main group centres is more challenging due to the resulting high stability of the M(*n*+2) oxidation state, particularly for the lighter, more earth abundant elements such as aluminium and silicon. This impedes the catalytic ability of main group metals in traditional redox based cycles as turnover is not possible. This also applies to main group chemistry in general, however alternative catalytic processes have been utilised which circumvent the change in oxidation state. These non‐redox processes typically involve a series of σ‐bond metathesis/insertion steps to enable turnover.^[16]^

**Scheme 1:**
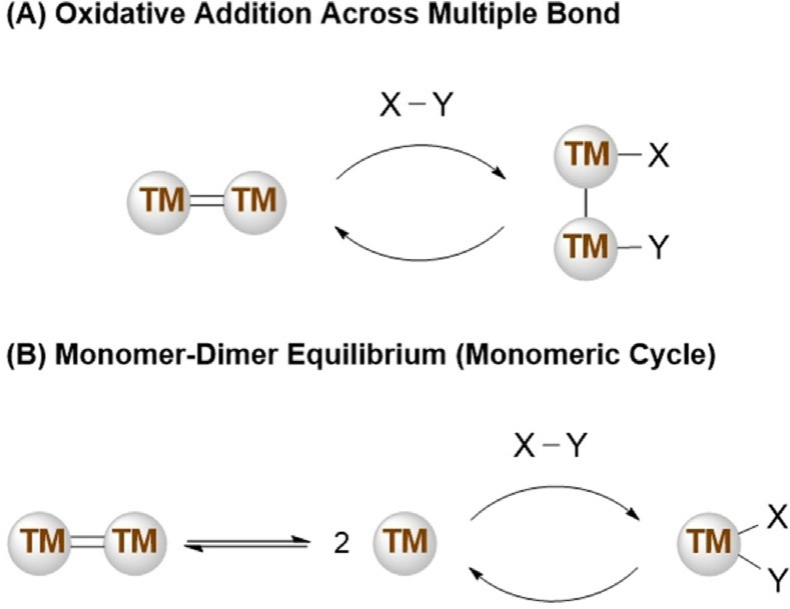
Generic catalytic cycles for the activation of substrates (X–Y) by transition metal multiple bonds.

Multiple bonds offer an attractive tool for the main group chemist due to the presence of a metal‐metal bond. Metal–metal bonds from across the periodic table bonds have enabled a series of unique bond activations and catalysis, particularly in transition metal chemistry^[17]^ and f‐elements.^[18]^ In transition metal chemistry, metal–metal bonds have broad applications and have been found to play key roles in catalytic processes.^[17]^ The use of transition metal multiple bonds in catalysis has allowed for retention of the dinuclear complex on addition of the substrate (Scheme 1 A). They have also found roles as pre‐catalysts, providing access to a monomeric low valent “active” species (Scheme 1 B).

Both above‐mentioned steps within a catalytic process could be envisioned for main group multiple bond chemistry, yet their use in catalysis is currently limited to just two examples. A digermyne, a germanium‐germanium triple bond, which was used for the cyclotrimerisation of terminal alkynes^[19]^ and a dialumene, an aluminium–aluminium double bond, which was found to be an active pre‐catalyst for the reduction of carbon dioxide.^[20]^ These two examples show the potential for this field to offer an alternative to expensive and often toxic transition metals that are currently used in industry. In addition to main group multiple bonds, low oxidation state and/or coordinate main group complexes have also undergone a renaissance in recent years. With several new breakthroughs revealing new classes of compounds, such as nucleophilic aluminyls,[^21^, ^22^] as well as advances in bond activations and catalysis.[^23^, ^24^, ^25^, ^26^, ^27^, ^28^] The synthesis and bonding nature of main group multiple bonds have been reviewed recently.[^5^, ^6^, ^29^, ^30^, ^31^, ^32^, ^33^] This minireview, therefore, focusses on the reactivity of these compounds highlighting the unique transformations that can be achieved due to molecular main group multiple bonds.

## Group 13 Multiple Bonds

### E^13^–E^13^ multiple bonds

**Figure 3:**
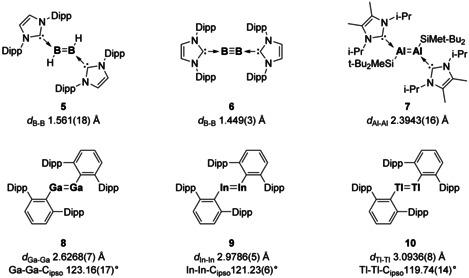
Selected examples of E^13^–E^13^ multiple bonds. Dipp=2,6‐di‐*iso*‐propylphenyl.

Historically it was thought that group 13 elements (E^13^) would preclude multiple bond formation. The presence of only 3 valence electrons, as well as weak E^13^−E^13^ bond energies, leads to a high tendency for decomposition and disproportionation reactions. One successful method to overcome these challenges is to use Lewis bases to help stabilise the multiple bond, through donation of a lone pair into the vacant E^13^ p‐orbital. Owing to their easily tuneable steric and electronic properties, NHCs have a proven track record in main group chemistry,^[34]^ and have enabled the isolation of the first diborene (**5**),^[35]^ diboryne (**6**)^[8]^ and dialumene (**7**)^[7]^ (Figure 3). As expected for lighter elements these complexes exhibit planar geometries. On descending the group, the stability of the lone pair increases, and Lewis base stabilisation is no longer required. The three‐coordinate *trans*‐bent double bonds can be stabilised using sterically demanding terphenyl ligands (Compounds **8**–**10**, Figure 3).[^36^, ^37^, ^38^] Recent reviews have highlighted the different synthetic methodologies and alternate ligand choices for the formation of these electron precise multiple bonds, and as such will not be discussed herein.[^29^, ^39^, ^40^]

**Scheme 2:**
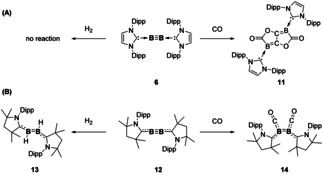
Ligand controlled small molecule activation with diborynes.

In terms of their reactivity, homonuclear E^13^ bonds have found themselves to be efficient tools for small molecule activation. For the lightest element, the reactivity of diboryne compounds were found to be influenced by the π‐acceptor ability of the supporting ligand.[^41^, ^42^, ^43^] CO coupling was observed for NHC‐stabilised complex (**6**) however these were unable to activate dihydrogen (Scheme 2 a). Switching to cyclic alkyl amino carbene (cAACs) ligands, which have increased π‐acceptor abilities relative to NHCs, enables diboryne (**12**) activation of dihydrogen at room temperature (**13**) but only coordination of CO was observed (**14**, Scheme 2 b). Additionally, NHC‐stabilised diborenes are able to fixate CO_2_ via [2+2]‐cycloaddition. The CO_2_ fixated compound was found to be thermally unstable and rearranged at room temperature through C−O cleavage and loss of the B−B bond to form a bridging carbonyl species.^[44]^

**Scheme 3:**
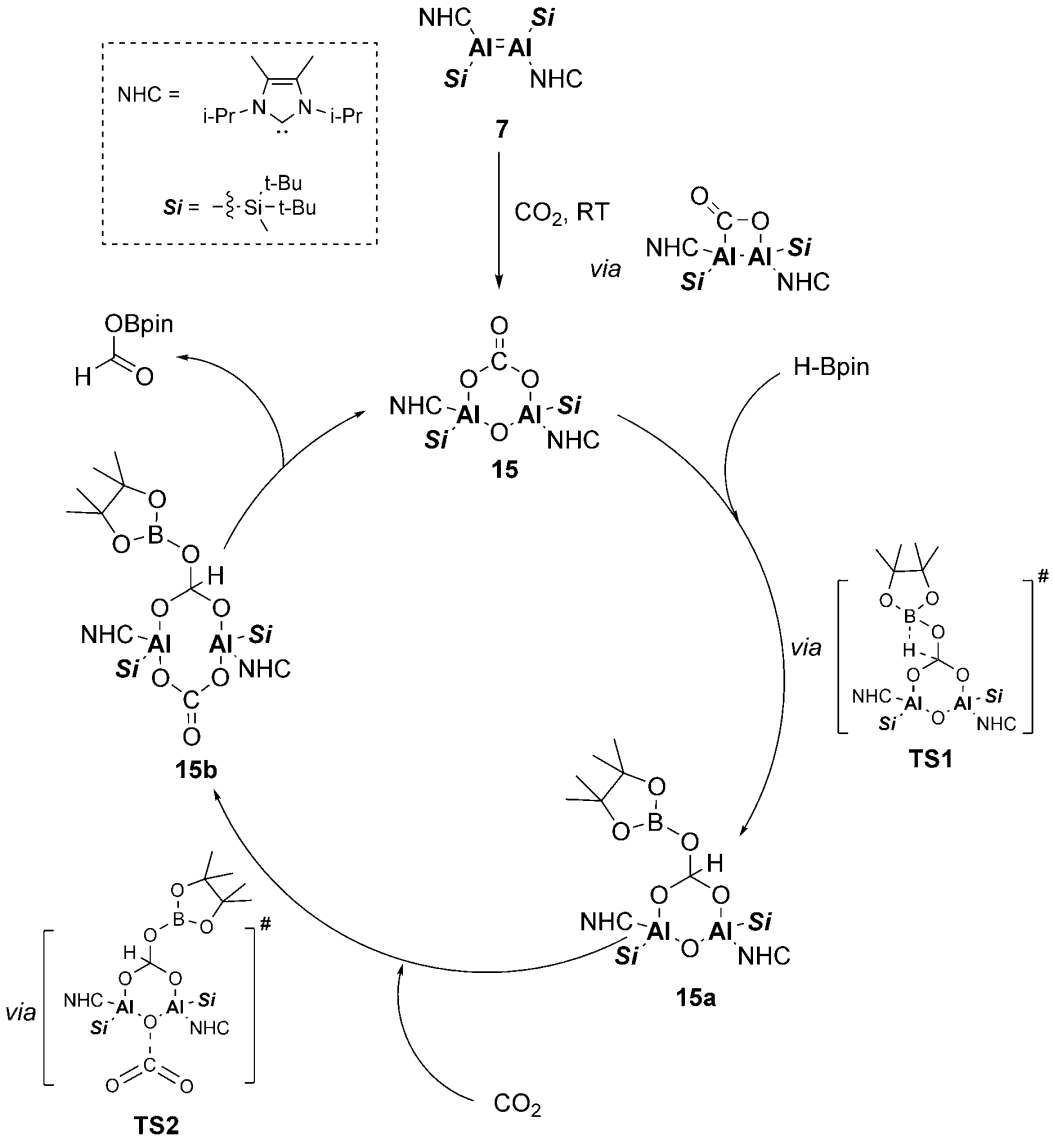
Proposed catalytic cycle for CO_2_ reduction by dialumene (**7**).

In a similar fashion, the NHC‐stabilised dialumene (**7**) was also found to fixate CO_2_.^[20]^ In contrast to the diborene system, the CO_2_ fixation product was found to be stable and underwent further reactivity in the absence (carbonyl formation) or presence (carbonate formation) of additional CO_2_. The ability to access this carbonate species was found to be pivotal in the catalytic reduction of CO_2_. Catalytic reduction could be achieved with the addition of pinacol borane (HBpin). The mechanism for this was probed computationally (Scheme 3). It was found that dialumene (**7**) acts as a pre‐catalyst which forms carbonate **15** upon addition of CO_2_. Reduction by HBpin occurs at the exocyclic carbonyl to yield **15 a**. CO_2_ then inserts into the bottom side of the Al–Al line of centres. The resultant eight‐membered ring (**15 b**) collapses with release of the formic acid equivalent, regenerating **15** in the process. Whilst this catalytic cycle does not contain an Al‐Al bond, the dinuclear complex remains intact due to bridging oxo and carbonate units and the ability of this system to alternate between reduction and insertion on the different sides of the Al–Al line of centres.

**Scheme 4:**
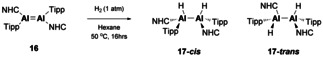
Dihydrogen activation by an aryl‐stabilised dialumene (**16**). Tipp=2,4,6‐tri‐*iso*‐propylphenyl.

Very recently, a second neutral NHC‐stabilised dialumene (**16**) was isolated.^[45]^ This differed to compound **7**, due to the use of an aryl supporting ligand, which resulted in a *trans*‐bent and twisted geometry. The influence of the different ligands on the dialumene geometry was largely the result of the steric demands of the ligand. In terms of the electronics, the silyl ligand results in an almost neutral Al_2_ core whilst the aryl dialumene core is highly polarised, which can be attributed to the relative differences in electronegativities. Notably, this results in a much more reactive dialumene and reactivity towards sterically more demanding substrates is now possible due to the increased flexibility in the *trans*‐bent and twisted structure. Furthermore, facile activation of dihydrogen is now achievable (previously **7** showed no reactivity), yielding both the *cis* and *trans*‐isomers of a 1,2‐dihydro‐dialumane (**17**, Scheme 4). The influence of ligand choice is further implicated in two different catalytic reactions, namely hydroboration of CO_2_ and amine‐borane dehydrocoupling. The aryl‐stabilised dialumene (**16**) is more catalytically active and results in different product distributions. Thus, indicating the likelihood of alternate mechanisms simply through change of supporting ligand.

Reactivity of heavier E^13^ multiple bonds (Ga‐Tl)[^36^, ^37^, ^38^] are limited to a few examples. Digallene (**8**) is capable of activating dihydrogen and ammonia^[46]^ whilst dithallene (**10**) readily dissociates to its monomeric form in hydrocarbon solutions and therefore acts as a Lewis base in the formation of donor‐acceptor complexes.^[38]^ Further reactivity studies of digallene show that whilst it can dissociate to its monomeric species it is in fact the double bond that is responsible for the observed reactivity.[^47^, ^48^, ^49^] The chemistry of homonuclear E^13^ multiple bonds is still in its infancy, with the potential for these systems far from fully realised. Boron is capable of forming stable triple bonds with itself, whilst aluminium^[50]^ and gallium^[51]^ have been isolated as anionic species, thus making their true bond order challenging to define. Extension to heteronuclear multiple bonding is also limited within E^13^. Currently, there is one example of a E^13^–E^13’^ multiple bond, a few examples of E^13^‐E^14^ and E^13^‐E^15^ multiple bonds and several E^13^–E^16^ multiple bonds. Again, this is due to difficulties within the synthesis and stabilisation of these inherently reactive species.

### E^13^–E^13′^ multiple bonds

**Scheme 5:**
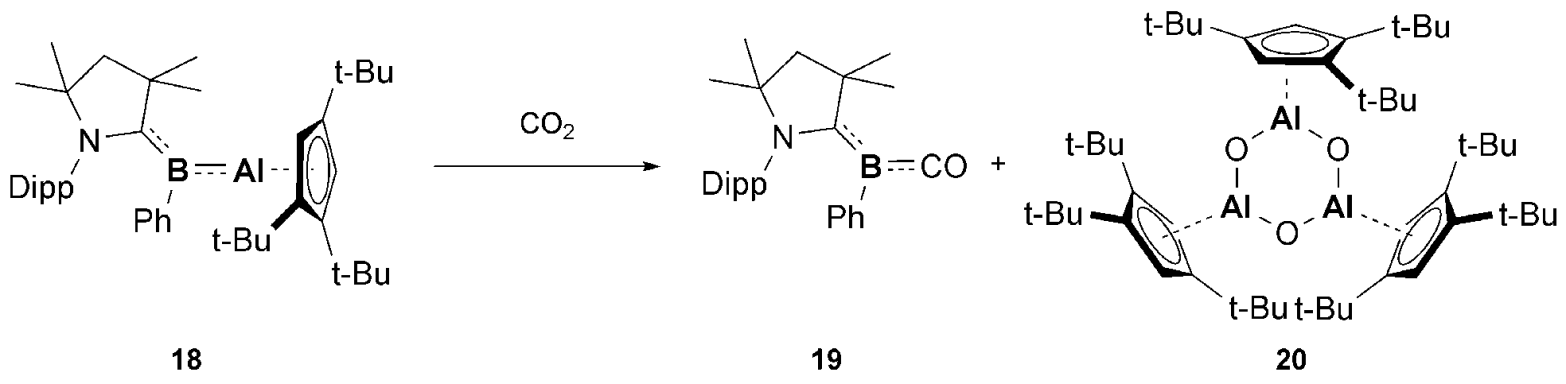
CO_2_ reduction by a boron‐aluminium multiple bond.

The first, and currently only, group E^13^‐E^13’^ complex which contains multiple bond character, was reported by Braunschweig and co‐workers (Compound **18**, Scheme 5).^[52]^ The resultant cAAC stabilised B‐Al bond is best described as a 3‐centre‐2‐electron π‐bond as DFT analysis found the HOMO to contain conjugation across the Al‐B‐cAAC unit. This bonding situation had been previously observed in related cAAC stabilised B−CO or B−N_2_ complexes.[^53^, ^54^] **18** was also found to be efficient in small molecule activation as reaction of **18** with CO_2_ resulted in C−O and B−Al cleavage to form a borylene CO complex and an aluminoxane, compounds **19** and **20**, respectively (Scheme 5).

### E^13^–E^14^ multiple bonds

**Scheme 6:**
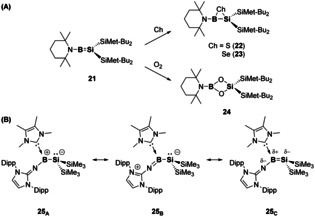
(a) Reactivity of borasilene (**21**) towards chalcogens. (b) Selected resonance structures of Lewis base stabilised borasilene (**25**).

Examples of group E^13^–E^14^ multiple bonds are also limited to a handful of examples and as such the reactivity of these compounds is largely unknown. Attempts to isolate borasilenes, that is compounds containing B=Si double bonds, have been achieved through matrix isolation techniques.^[55]^ To date, only one neutral borasilene (**21**)^[56]^ and one Lewis base stabilised borasilene (**25**)^[57]^ exist in the condensed phase. Reactivity studies of **21** towards chalcogens revealed the formation of three‐membered rings with sulfur (**22**) and selenium (**23**), whilst with oxygen (**24**) a four‐membered ring, with loss of the B−Si bond, was found (Scheme 6 a).^[58]^ The bonding situation in the Lewis base stabilised borasilene (**25**), based on experimental solid‐state structural features and DFT calculations, suggest **25** is best described as a zwitterionic double bond in contrast to borasilene **21**. A series of resonance structures can be drawn (Scheme 6 b) with **25_A_**, wherein the positive charge is located on the boron atom, representing the major resonance form. Attempts to use compound **25** for small molecule activation revealed no reactivity towards dihydrogen and an ill‐defined mixture with CO_2_. However, B−Si cleavage was observed on addition of HBpin, to yield BH_2_ and Si(Bpin)_2_ containing species.^[57]^

### E^13^–E^15^ multiple bonds

**Scheme 7:**
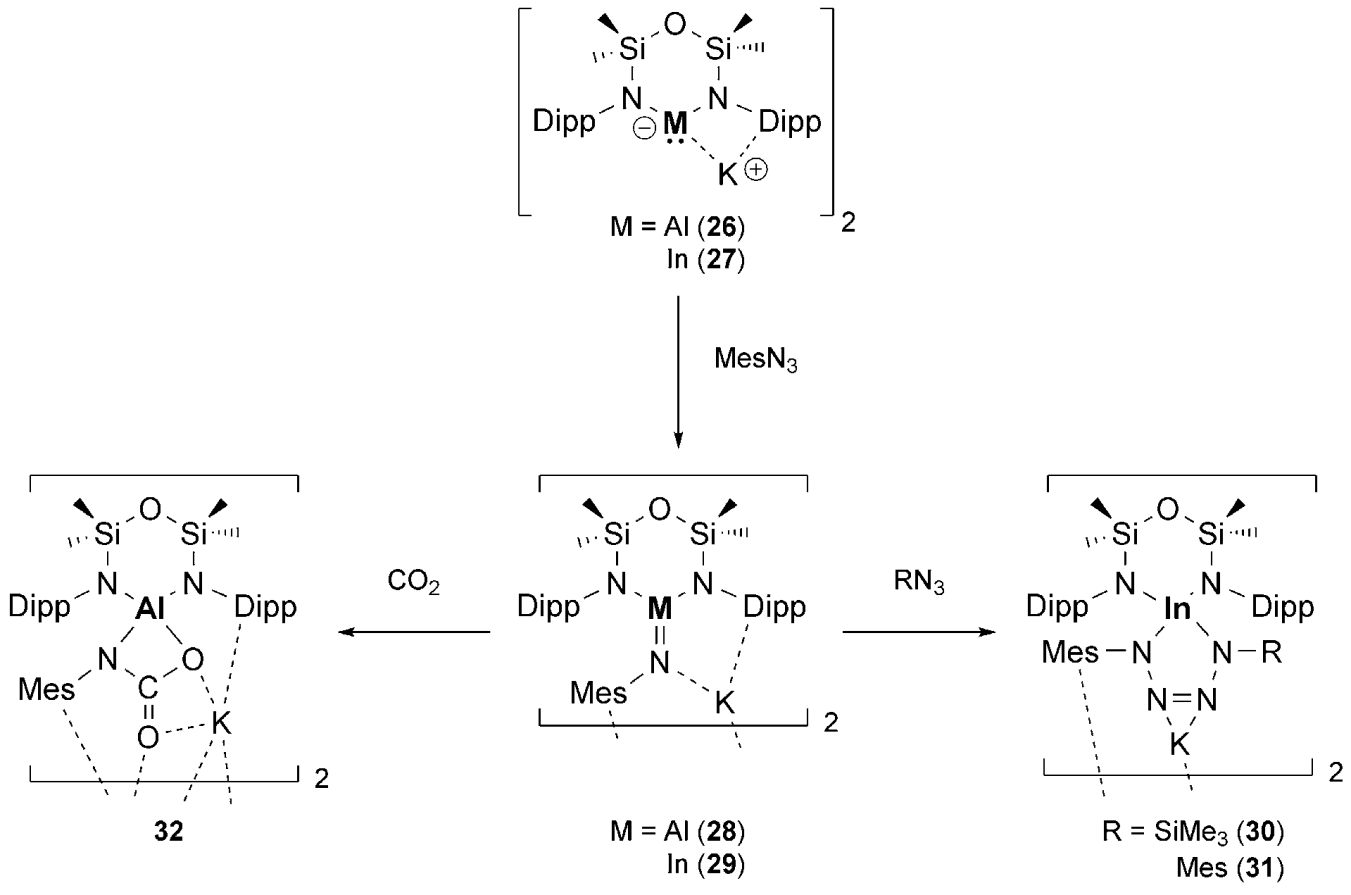
Reactivity of E^13^‐imides towards CO_2_ (Al only) and organic azides (In only).

E^13^–E^15^ (pnictogen) multiple bonds are of interest to both academia and industry due to their interesting materials properties. Boron nitrides are widely used in the ceramics industry due to their high thermal and chemical stability,^[59]^ whilst AlN, GaN, InN have interesting electronic properties.^[60]^ Attempts to isolate discrete M=NR complexes has found limited success. Due to the necessity of sterically demanding ligands, the M=NR moiety is kinetically protected and therefore the reactivity of these compounds is somewhat impeded. To date, amongst the structurally characterised examples of E^13^ imides (E^13^=NR, E^13^=Al,[^61^, ^62^, ^63^] Ga,[^64^, ^65^, ^66^] and In[^66^, ^67^]) only a few have reported further reactivity. Both Al and In imides (compounds **28** and **29**, respectively) were synthesised from the corresponding E^13^(I) nucleophiles (Al **26**; In **27**) on reaction with MesN_3_ (Mes=2,4,6‐tri‐methylphenyl) (Scheme 7). In terms of their reactivity, the indium analogue undergoes further reactivity with organic azides to yield four membered rings (Scheme 7, compounds **30**, **31**).^[67]^ Whilst the aluminium imide shows further reactivity with CO_2_ via a [2+2]‐cycloaddition to yield a carbamate dianion (Scheme 7, compound **32**).^[62]^

**Scheme 8:**
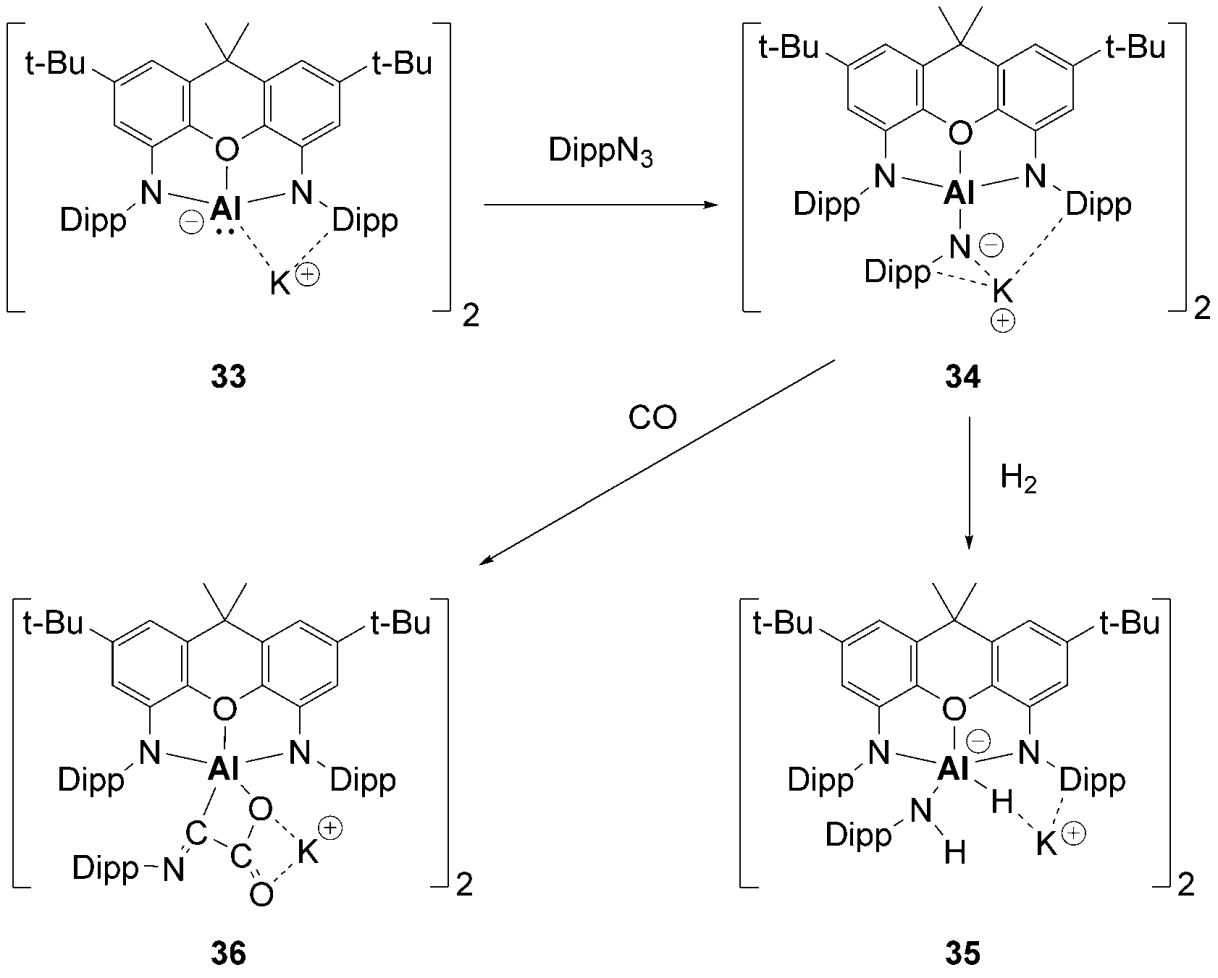
Synthesis and reactivity of an Al‐imide (**34**) towards small molecules.

In related work Aldridge, Goicoechea and co‐workers also obtained an Al imide complex (**34**) from reaction of their aluminyl ion (**33**) with DippN_3_ (Scheme 8).^[63]^ The highly polar nature of the Al−N bond was highlighted through its reactivity with small molecules. Dihydrogen was found to add in a 1,2‐fashion across the Al−N bond at elevated temperatures (80 °C) to yield an amido aluminium hydride complex (**35**). Whilst two molecules of CO were found to react with **34** to yield compound **36** which is the result of C−O cleavage and C−C bond formation.^[63]^

**Scheme 9:**
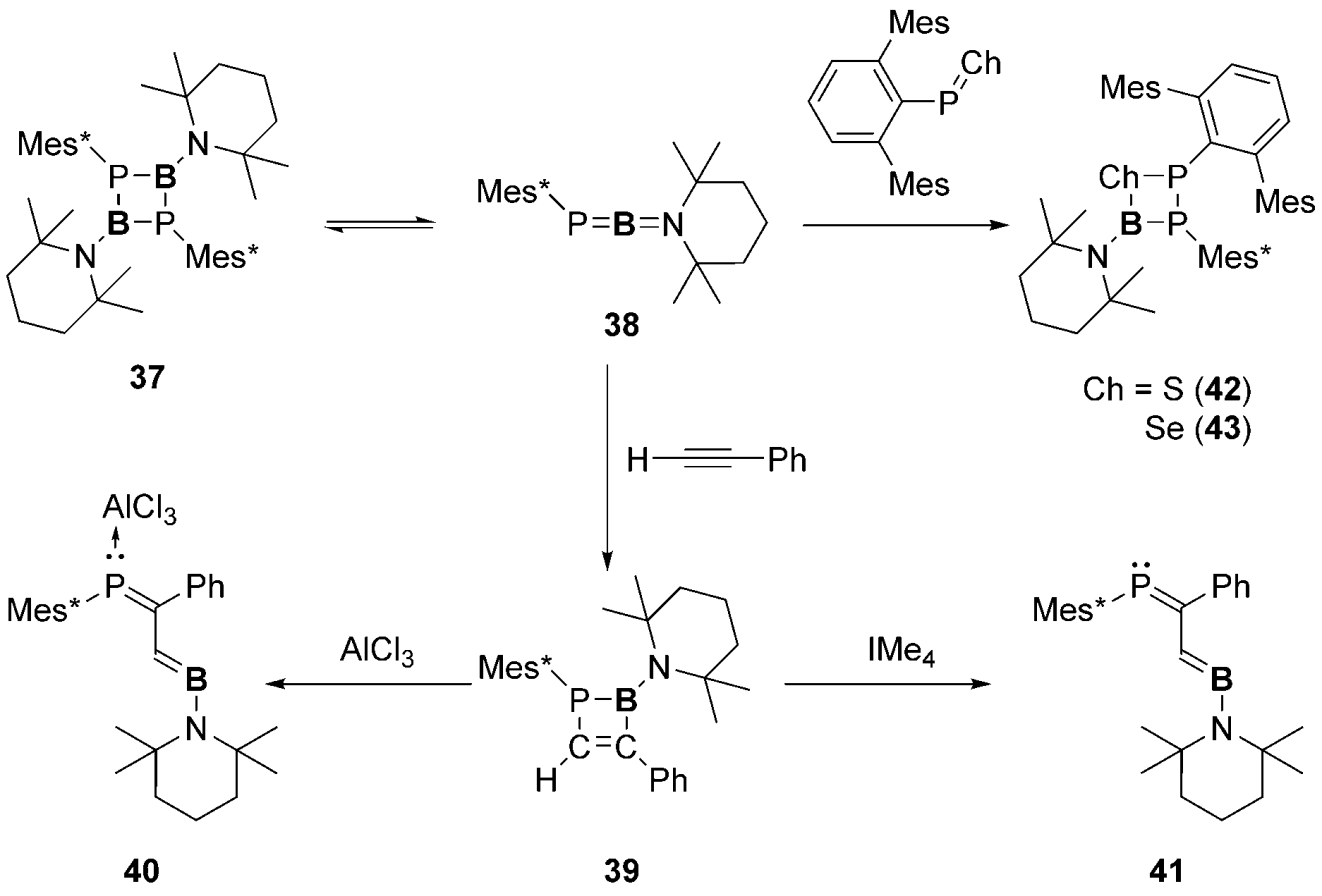
Phosphaborenes as a synthetic reagent. Mes=2,4,6‐trimethylphenyl; Mes*=2,4,6‐tri‐*tert*‐butylphenyl. IMe_4_=1,3,4,5‐tetramethyl‐imidazol‐2‐ylidene.

Descending further down the pnictogen series, a few examples of Lewis acid or based stabilised B=P bonds exist, as well as B=As bonds.[^68^, ^69^, ^70^, ^71^] Reactivity of these multiple bonds is scarce, but they have shown that they can be used as reagents to access C−C/P−B isoteres (Scheme 9).^[72]^ Compound **37** was found to dissociate at elevated temperatures to provide the phosphaborene (**38**) in solution, this then undergoes [2+2]‐cycloaddition with phenylacetylene to yield compound **39**. The ring opening reaction can be promoted through use of Lewis acids and bases to yield compounds **40** and **41**, respectively.^[72]^ Further reactivity of **38** showed that it could be used to access mixed main group element rings (Compounds **42** and **43**, Scheme 9).^[73]^

### E^13^–E^16^ multiple bonds

**Figure 4:**
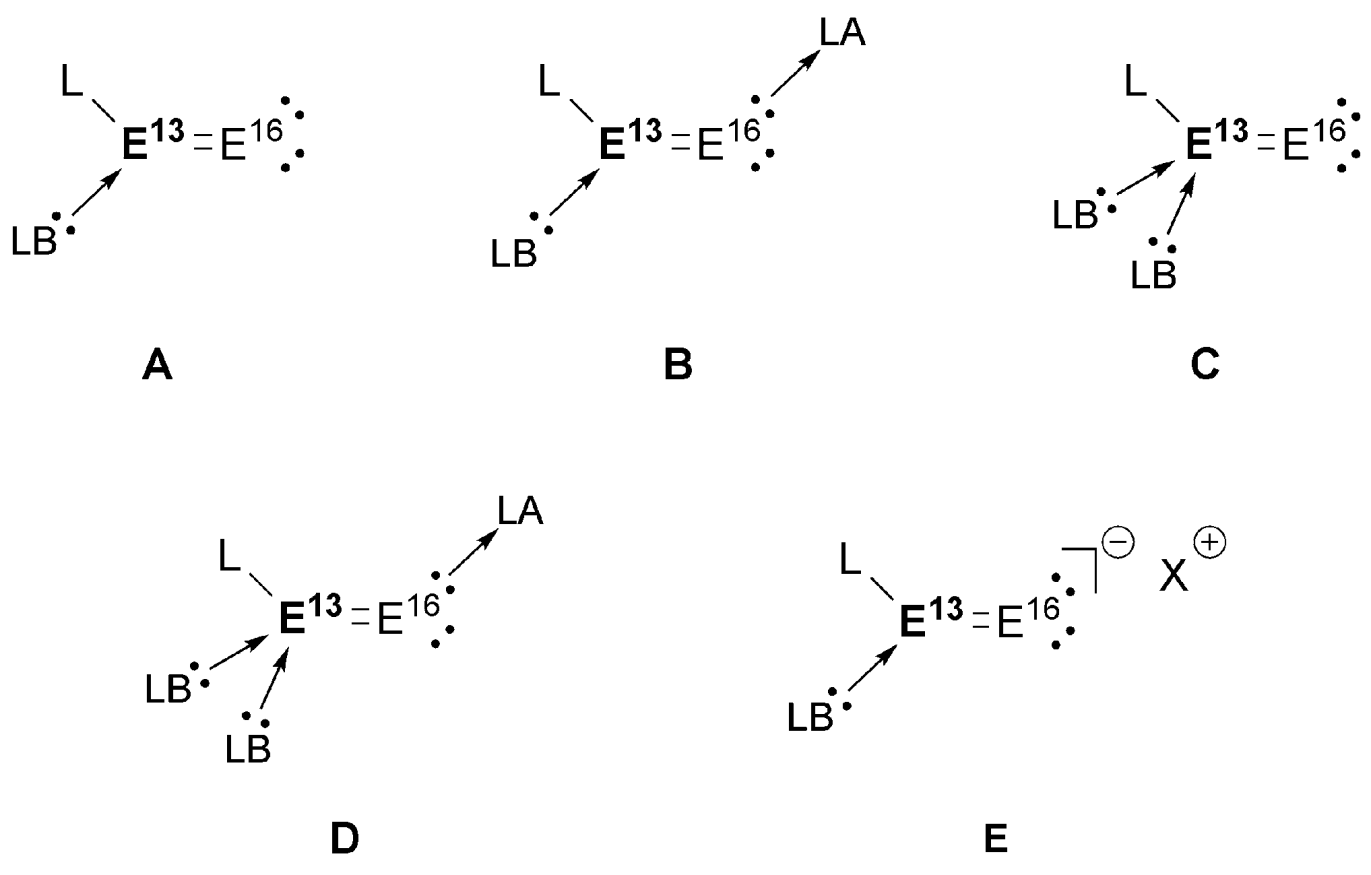
Different Lewis acid and base strategies for stabilisation of terminal E^13^=E^16^ multiple bonds.

E^13^–E^16^ (chalcogen) bonds are also of high interest due to their materials properties. For example, alumina has found widespread use in industry from heterogenous supports to materials and even cosmetics.^[74]^ The inert nature of alumina arises from the large differences in electronegativities (Al 1.61, O 3.44) which results in a thermally stable material with high electrical resistance. The highly polarised bonds, however, also increase the difficulty of isolating a discrete E^13^=E^16^ multiple bond. As such, additional Lewis acid and base stabilisation is often required to stabilise the terminal E^13^=E^16^ bond (Figure 4).

**Scheme 10:**
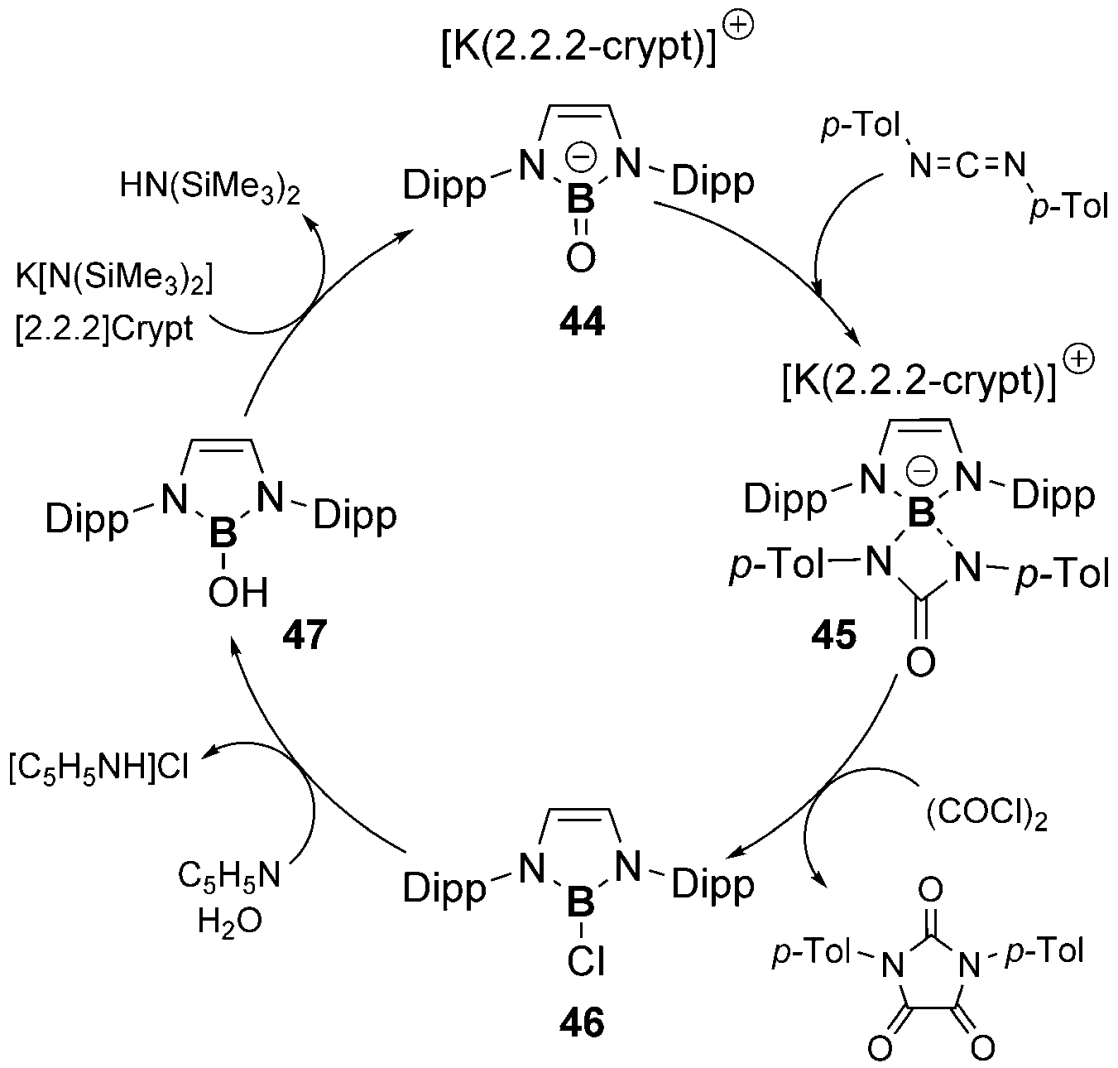
Step‐wise synthetic cycle for oxide ion transfer agent, **44**.

The synthesis and isolation of E^13^–E^16^ multiple bonds have been highlighted recently,[^40^, ^75^] and as such only recent progress in terms of their reactivity will be discussed herein. There have been significantly more reports of boron multiple bonds to chalcogens than any other E^13^ elements, including the only example of E^13^≡E^16^ triple bond.^[76]^ Aldridge and co‐workers, recently reported the isolation of anionic oxoborane (**44**),^[77]^ which is stabilised akin to type **E** in Figure 4. This compound can undergo π‐bond metathesis with CS_2_ to yield the related anionic thioxoborane. Furthermore, **44** was shown to act as an oxygen transfer agent (Scheme 10). Utilising a similar approach to Betrand, where the isoelectronic phosphinonitrene can act as a nitrogen transfer agent,^[78]^ 1,3‐di‐*p*‐tolylcarbodiimide was added to **44** to yield **45**. Addition of oxalyl chloride released the functionalised cyclic urea derivative with concomitant formation of the boron‐chloride species (**46**). Compound **44** could then be regenerated in a step wise manner through conversion to the boronic acid species (**47**), followed by deprotonation in the presence of a K‐sequestering cryptand ligand (Scheme 10).^[77]^

**Scheme 11:**
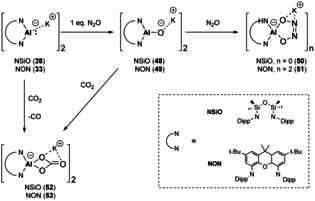
Synthesis and reactivity of monoalumoxanes.

A similar approach to the stabilisation of Al−O bonds was undertaken (Figure 4, type **E**). Again starting from the corresponding anionic Al^I^ nucleophiles (**26** and **33**), reaction with 1 equiv. of N_2_O results in the formation of monoalumoxane anions (Scheme 11, **48** ^[79]^ and **49** ^[80]^). These are thought to contain some multiple bond character however, it is dominated by the anionic resonance form.^[79]^ In the presence of additional N_2_O, five membered heterocycles are formed (**50** and **51**). These can either be isolated from the stepwise approach (via compounds **48** and **49**) or via reaction of the nucleophilic starting materials (**26** and **33**) with excess N_2_O. A similar strategy can also be employed to access aluminium carbonate species (**52** and **53**), direct reaction of excess CO_2_ with the Al^I^ nucleophiles results in carbonate formation with loss of CO. Also, the reaction of monoalumoxanes (**48** and **49**) with CO_2_ results in the direct insertion of the C−O bond into Al−O bond to yield the corresponding carbonates (**52** and **53**). Sequestering the potassium ion in **48** with [2,2,2‐cryptand], resulted in formation of a hydroxy species due to C−H activation of the iso‐propyl methyl of the flanking aryl group. Whilst reaction of **49** with dihydrogen yields an aluminium hydride hydroxy complex. This indicates the high Lewis basicity of the oxide anion in alumoxanes.

**Scheme 12:**
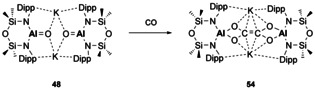
Synthesis of ethenetetraolate ligand (**54**).

The reactivity of **48** towards CO was examined to provide some insight into formation of an ethenetetrathiolate species, which was obtained from the reaction of **26** with CS_2_.^[81]^ It was postulated that use of **48** would allow for isolation of intermediates due to the increased Al−O bond strength in comparison to Al−S (bond dissociation energies (BDE): 501 kJ mol^−1^ vs. 332 kJ mol^−1^, respectively). Addition of CO to **48** resulted in C−C bond formation in the form of the analogous ethenetetraolate ligand (**54**, Scheme 12). Mechanistic insights were provided by DFT calculations and highlighted the importance of the potassium counterion in this reaction, as several stabilising interactions from K^+^ were found in this transformation.^[81]^

**Scheme 13:**
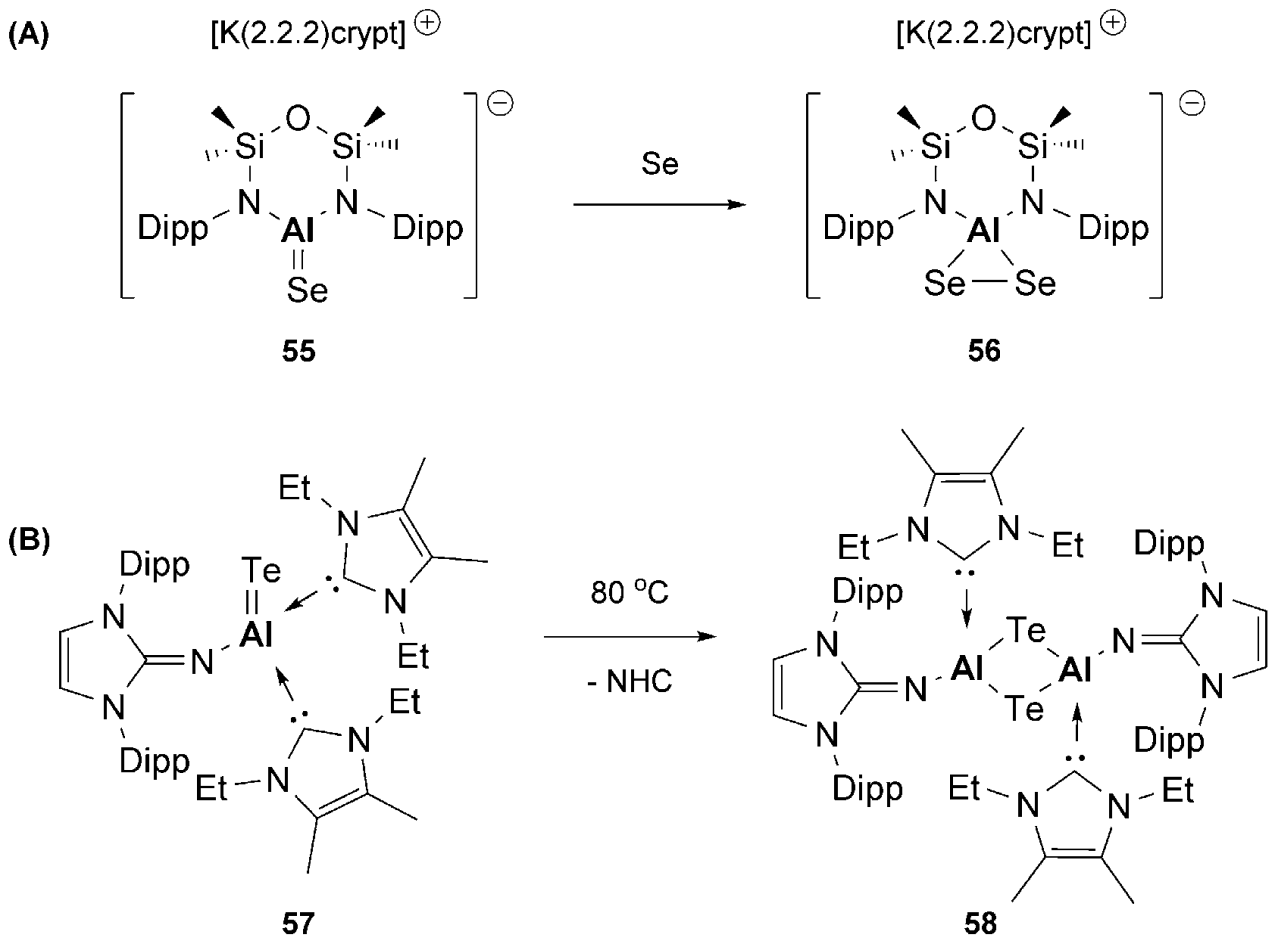
Reactivity of heavier aluminium chalcogenides.

Reactivity studies with the heavier aluminium chalcogenides is limited. Coles reported the selenium analogue to **48**, which was synthesised in the presence of a cryptand ligand to sequester the cation (**55**, Scheme 13 a),^[82]^ whilst in the absence of the cryptand a polymeric Al−Se species was obtained. Addition of a second equivalent of selenium to **55** affords a planar three‐membered AlSe_2_ ring (**56**). Evidence for the high degree of polarity in E^13^‐E^16^ bonds is shown by the Lewis base stabilised Al=Te complex (**57**).^[83]^ This monotopic compound readily dimerises to **58**, with loss of one Lewis base, on mild heating (Scheme 13 b). Whilst there are a few examples of Ga and In chalcogenide multiple bonds,[^84^, ^85^, ^86^, ^87^] no onwards reactivity of these compounds has been reported. Heavier E^13^–E^16^ organometallic containing complexes, however, find themselves to be useful single source precursors for materials applications.^[60]^

## Group 14 Multiple Bonds

### E^14^–E^14^ multiple bonds

**Figure 5:**
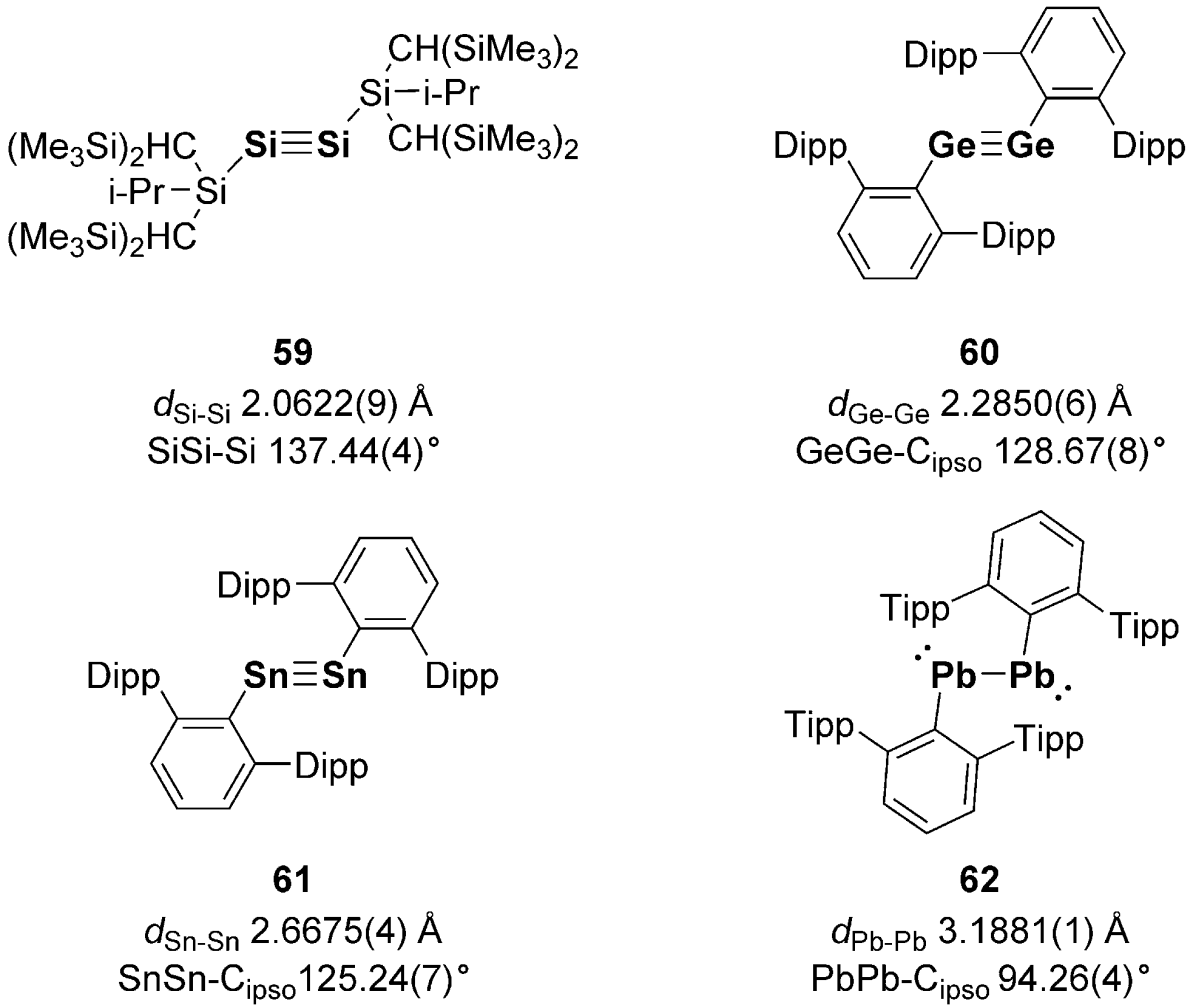
First reported examples of E^14^–E^14^ triple bonds. Dipp=2,6‐di‐*iso*‐propylphenyl. Tipp=2,4,6‐tri‐*iso*‐propylphenyl.

Of the main group multiple bonds, E^14^ elements are the most widely studied. For the lightest E^14^ member, carbon, its multiple bonds with itself and other elements account for the majority of known multiple bonds in existence, both those occurring naturally and synthetically.^[88]^ In contrast, heavier E^14^ multiple bonds have only come to fruition in the last 40 years, starting with West's disilene.^[1]^ Since then a plethora of E^14^=E^14^ double bonds have been isolated and been the subject of numerous reviews.[^5^, ^6^, ^29^, ^89^, ^90^, ^91^, ^92^] With their 4 valence electrons, triple bond formation is much more facile in comparison to E^13^ and disilynes (**59**),^[93]^ digermynes (**60**),^[94]^ distannynes (**61**)^[95]^ and diplumblynes (**62**)^[96]^ have all been isolated (Figure 5). Here, the lone pair effect on *trans*‐bent geometries is clearly observed as alkynes are linear whilst diplumbynes bear R−Pb−Pb angles of nearly 90°, and are therefore better described as diplumbylenes with a formal single bond and active lone pairs on each lead centre.^[96]^

**Scheme 14:**
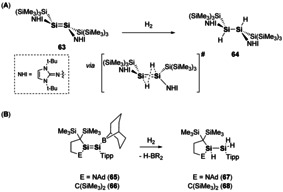
(a) Selective *anti*‐addition of dihydrogen to a highly *trans*‐bent and twisted disilene (b) Ligand controlled activation of dihydrogen.

Homodiatomic E^14^ multiple bonds have been shown to react with a number of substrates including small molecules.^[97]^ It was the latter that first drew the comparisons to transition metals,[^14^, ^15^] as digermyne showed ambient temperature reactivity towards dihydrogen.^[98]^ Even though disilenes are arguably the most studied E^14^ multiple bond, it was only recently that dihydrogen activation was achieved.^[99]^ A highly *trans*‐bent and twisted disilene (**63**) was isolated, which is stabilised by sterically demanding *N*‐heterocyclic imine (NHI) ligands and hypersilyl (hypersilyl=Si(SiMe_3_)_3_) groups. Disilene **63** contains a long Si–Si double bond (2.3134(7) Å, average ≈2.22 Å), so can be best described as a weak double donor‐acceptor bond. On reaction with dihydrogen (1 bar) complete loss of the characteristic purple colour of **63** was observed within 10 minutes, this resulted in the formation of 1,2‐disilane (**64**, Scheme 14 a).^[99]^ Notably, **64** is the result of *anti*‐addition, in contrast to alkene hydrogenation where *syn*‐addition is favoured. This experimental observation was explained through computational analysis as the staggered ligand arrangement reduces the stability, whilst pre‐organising the central Si=Si bond for concerted *anti*‐addition.^[99]^ Disilene **63** has also been shown to react with other small molecules such as NH_3_, CO_2_ and O_2_.^[100]^

Further examination of disilene dihydrogen activation was reported by Iwamoto.[^101^, ^102^] This found that choice of stabilising ligand was key, as a π‐accepting boryl group was required in order to achieve cleavage of dihydrogen (**65** and **66**). When the boryl group was replaced with an alkyl substituent (*i*Pr) no reactivity was observed. The rate of reaction could be further enhanced by use of a push‐pull disilene with a π‐donating amino substituent (**65**, Scheme 14 b).[^101^, ^102^]

**Scheme 15:**
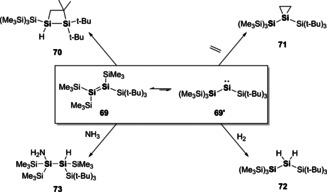
Reactivity at the disilene‐silylsilylene equilibrium.

Other notable recent advances in disilene chemistry has focussed on the disilene‐silylsilylene equilibrium (R_2_Si=SiR_2_↔R_3_Si=SiR). This equilibrium has been previously inferred to explain unexpected reaction products and thermally induced rearrangements. For example, Inoue and co‐workers reported the formation of a tetrasilyldisilene (**69**) which was proven to exist as the disilene in solution but largely reacted as a silyl silylsilylene (**69′**, Scheme 15).^[103]^ On leaving a solution of **69** at room temperature, C−H activation of the *t*Bu group of bis(silyl)silylene (**69′**) is obtained to yield **70**. [2+1]‐cycloaddition of ethylene occurs from **69′**, rather than [2+2]‐cycloaddition of **69**, to form **71**. Dihydrogen activation was possible due to the ability to access **69**′, whereas addition of NH_3_ occurs at the disilene (**69**) to yield the hydroaminated species **73** (Scheme 15).^[103]^

**Scheme 16:**
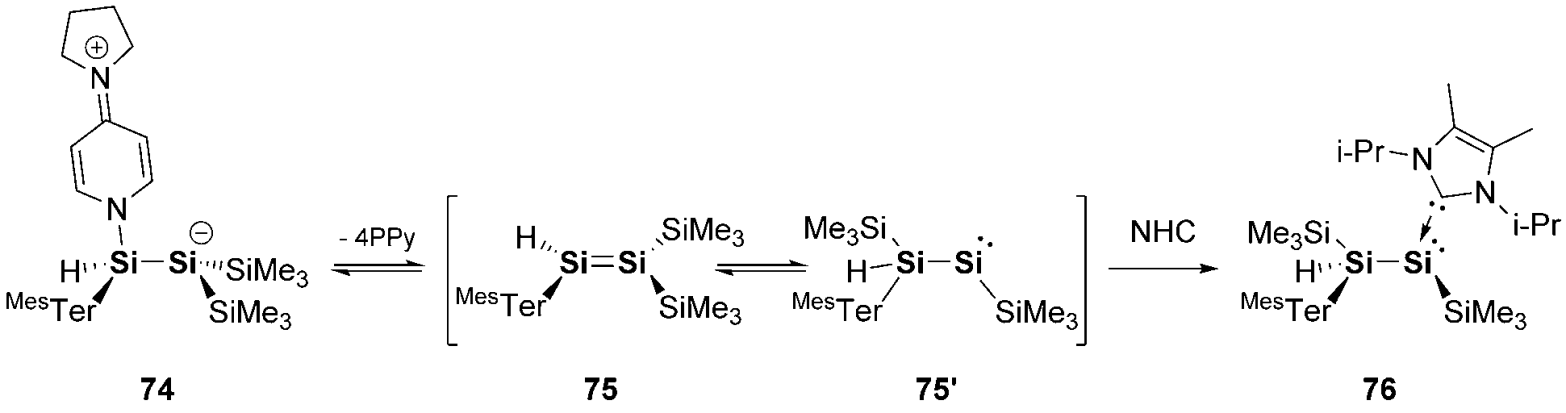
Intercepting the disilene‐silylsilylene equilibrium. ^Mes^Ter=2,6‐bis(2,4,6‐trimethylphenyl)phenyl.

Cowley and co‐workers found direct evidence for this disilene‐silylsilylene equilibria using a base‐coordination strategy. 4‐pyrollidinopyridine (4‐PPy) allowed for isolation of disilene **74**. 4‐PPy was found to be labile and in the presence of excess NHC ligand, isomerisation to silylsilene **76** was achieved (Scheme 16).^[104]^ This observation of **74** and **76** serves as direct evidence for the transient nature of **75** and **75′**, which is also supported by computational studies.^[104]^ The ability to control this equilibrium provides a new route to access to the more reactive silylsilylene species. Two‐coordinate acyclic silylenes are highly reactive species as they contain a vacant coordination site and a lone pair, and as such have shown facile bond activations towards small molecules and a variety of substrates.[^24^, ^28^, ^105^, ^106^, ^107^] It could be envisaged that new catalytic cycles, based on the ability to control this equilibrium, could be achieved in a somewhat similar fashion to that depicted in Scheme 1 B. Wherein the multiple bond (disilene) is off cycle and the “active” species is the low valent main group centre (silylsilylene).

**Scheme 17:**
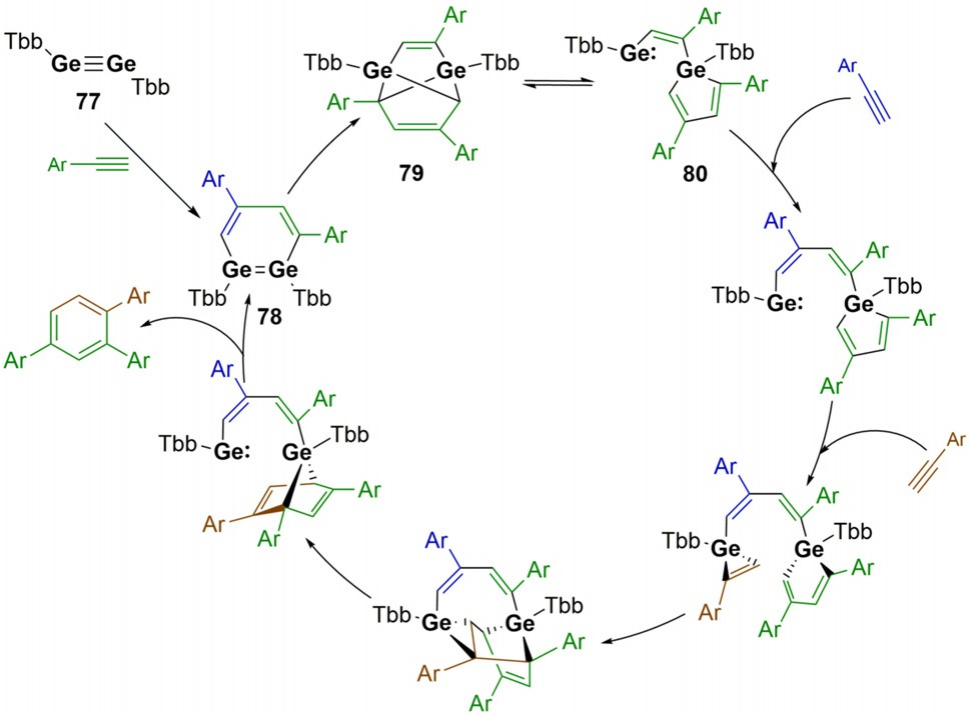
Digermyne catalysed cyclotrimerisation of alkynes. Tbb=4‐*t*Bu‐2,6‐[CH‐(SiMe_3_)_2_]‐C_6_H_2_.

The first example of a main group multiple bond being employed in catalysis was reported by Sasamori and co‐workers. Digermyne **77** was found to be active in the catalytic trimerisation of a range of phenylacetylenes to yield regioselective 1,2,4‐triarylbenzenes (Scheme 17).^[19]^ This reaction is specific to terminal arylacetylenes, as only stoichiometric reactions were observed with other unsaturated C−C bonds.[^108^, ^109^] The key to enabling turnover with arylacetylenes is the proposed equilibrium that exists between compounds **79** and **80**. Calculated energy barriers suggest that this is accessible at the higher temperatures (60 °C) at which the catalysis is performed. This equilibrium allows for access to the germole–germylene species (**80**) which contains the low valent Ge^II^ centre with a vacant coordination site for further substrate binding. The regioselectivity of this reaction is likely determined through the subsequent [1+2]‐cycloaddition and intramolecular [4+2]‐cycloaddition steps before release of the product and regeneration of digermene **78**. It is proposed that digermyne **77** serves as a pre‐catalyst to this transformation with **78** as the resting state.^[19]^

Advances in reactivity for the heaviest E^14^–E^14^ multiple bonds, that is, Sn and Pb, is somewhat hampered by their weak E^14^–E^14^ bonds and therefore tendency to dissociate in solution.[^92^, ^110^] Recent studies by Power and co‐workers showed the reversibility of distannyne–stannylene in toluene solutions^[111]^ as well as the reversibility of dihydrogen activation by distannynes.^[112]^ Despite diplumbylenes being the first example of a heavier E^14^–E^14^ triple bond, it was only recently that further examples emerged.^[113]^ A combined experimental and theoretical study found that London dispersion forces^[31]^ were important in the stabilisation of diplumbylenes. Those which are less *trans*‐bent contain increased multiple bond character compared with their more *trans*‐bent counterparts (i.e. closer to 90°).^[113]^

### E^14^−E^14′^ multiple bonds

**Figure 6:**
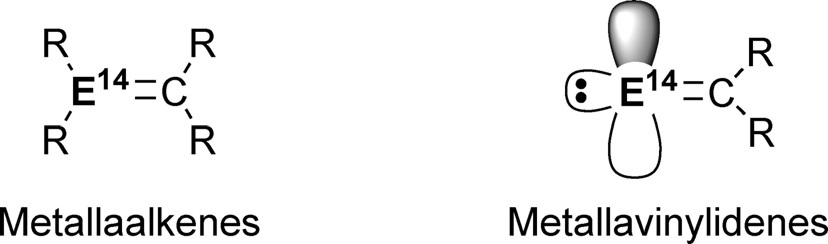
Different types of E^14^=C double bonds.

Since Brook first reported the isolation of a silene,^[3]^ several examples of metallaalkenes (R_2_E^14^=CR_2_, Figure 6) have been reported.[^5^, ^6^, ^114^, ^115^, ^116^, ^117^] The reactivity of these metallaalkenes have largely focussed on cycloaddition reactions of carbonyls and alkynes, where they have been found to follow the Woodward–Hoffman selectivity rules.^[118]^ Other E^14^=C containing species are metallavinylidenes (:E^14^=CR_2_, Figure 6) where the terminal E^14^ contains both an empty *p*‐orbital and a lone pair. In general, these compounds are reactive intermediates and require Lewis base stabilisation, however they have been shown to be ambiphillic in nature.^[119]^

**Scheme 18:**
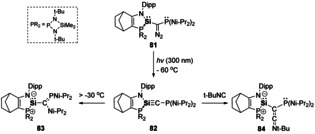
Synthesis and reactivity of a silyne (Si‐C triple bond).

Extension towards E^14^‐C triple bonds have also been achieved, both Ge^[120]^ and Sn^[121]^ derivatives were evidenced as transient species upon photolysis of their corresponding diazomethanes. Using a similar strategy, Kato and Baceiredo were able to isolate a base‐stabilised silyne (**82**, Scheme 18).^[122]^ This compound is stable up to −30 °C, however, above this temperature it undergoes a 1,2‐migration of the supporting ligand to form a phosphaalkene (**83**, Scheme 18). Furthermore, the carbenic character of **82** was shown via trapping with *tert*‐butyl isocyanide to form a keteneimine (**84**, Scheme 18).^[122]^

**Scheme 19:**
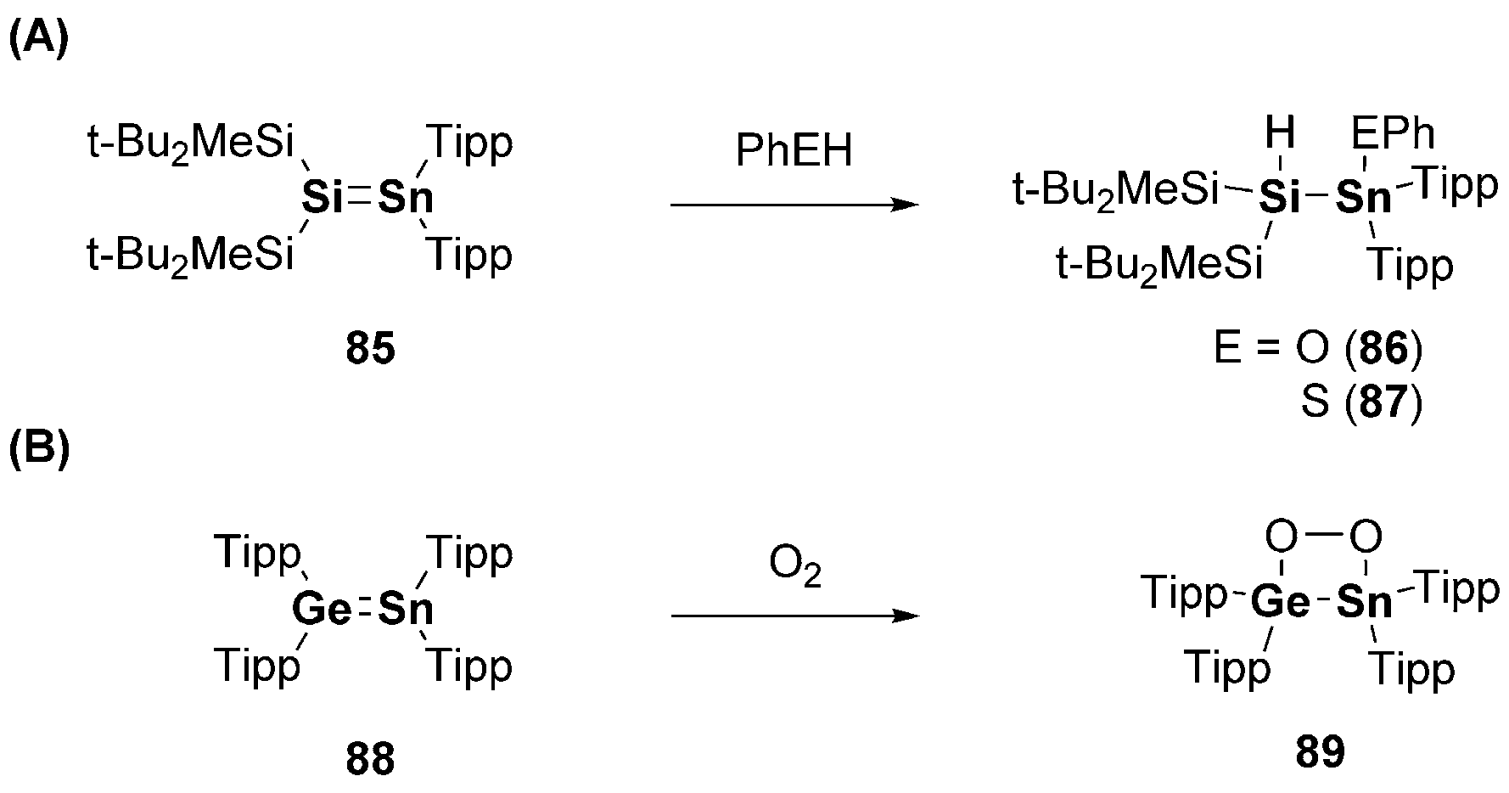
(a) Reactivity of silastannenes and (b) reactivity of germastannenes. Tipp=2,4,6‐tri‐*iso*‐propylphenyl.

The heavier mixed alkenes (i.e. E^14^=E^14′^, where E=Si, Ge, Sn) are proposed to have similar π‐bond strengths to their corresponding homo‐diatomic multiple bonds (e.g. Ge=Ge and Ge=Si are similar).^[123]^ Whilst the synthesis and reactivity of metallaalkenes (E^14^=C) is rather well established, the heavier analogues are rare in comparison.[^6^, ^114^] An example of a silastannene (**85**) was found to react according to the polarity of the double bond with phenols and thiophenols (Scheme 19 a).^[124]^ Whilst the reaction of dioxygen with germastannene (**88**) results in coordination and formation of a 4‐membered ring (**89**, Scheme 19 b).^[125]^

Other examples of mixed E^14^ multiple bonds are found within small inorganic ring systems^[126]^ and even as heteroallenes (R_2_E^14^=E^14^=E^14^R_2_).[^127^, ^128^] Although the latter complexes may be better described as containing a zerovalent central atom which is supported by coordinated tetrylenes (i.e. R_2_E^14^→E^14^←E^14^R_2_).[^32^, ^129^]

### E^14^–E^15^ multiple bonds

**Scheme 20:**
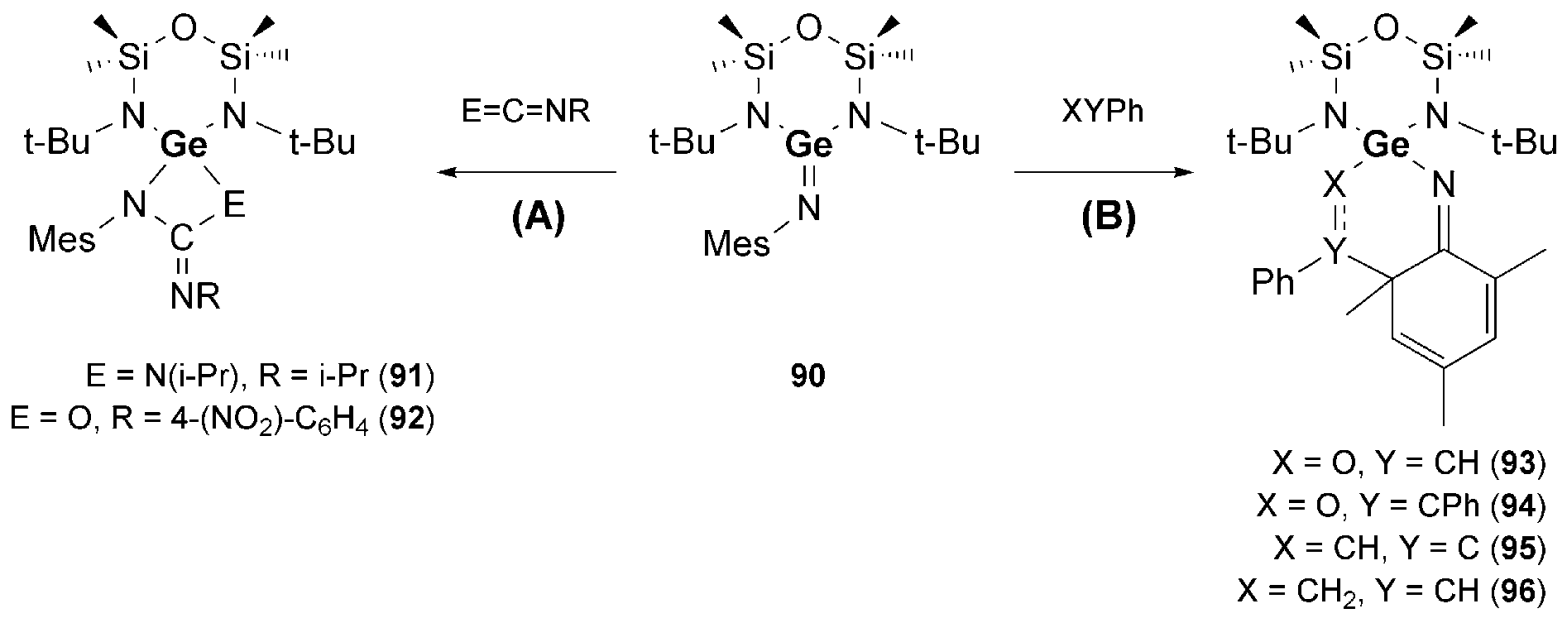
Metalloid behaviour of a germanimine (**90**).

It was not until 1981 that Becker reported the first example of a heavier E^14^−E^15^ bond (C≡P, phosphaalkyne).^[4]^ Since this report, many examples of phosphaalkenes have emerged and, as such, are beyond the scope of this article.[^130^, ^131^] Additionally, a range of silaimines have been isolated, which show increased reactivity compared to their imine counterparts. Their reactivity is also well documented and therefore will not be discussed herein.[^132^, ^133^, ^134^, ^135^, ^136^] Heavier imine analogues (Ge, Sn)[^6^, ^137^, ^138^] have also been isolated although are much rarer in contrast to silaimines. A recent study by Fulton and co‐workers showed that reactivity of a germanimine (**90**) exhibits “metalloid” type behaviour. The reactivity of **90** resembles that of transition metal imido complexes on reaction with heterocumulenes ([2+2]‐cycloaddition to form compounds **91** and **92**, Scheme 20 a), and that of imines as Diels–Alder reactivity was also observed ([4+2]‐cycloaddition, compounds **93**–**96**, Scheme 20 b).^[139]^

**Scheme 21:**
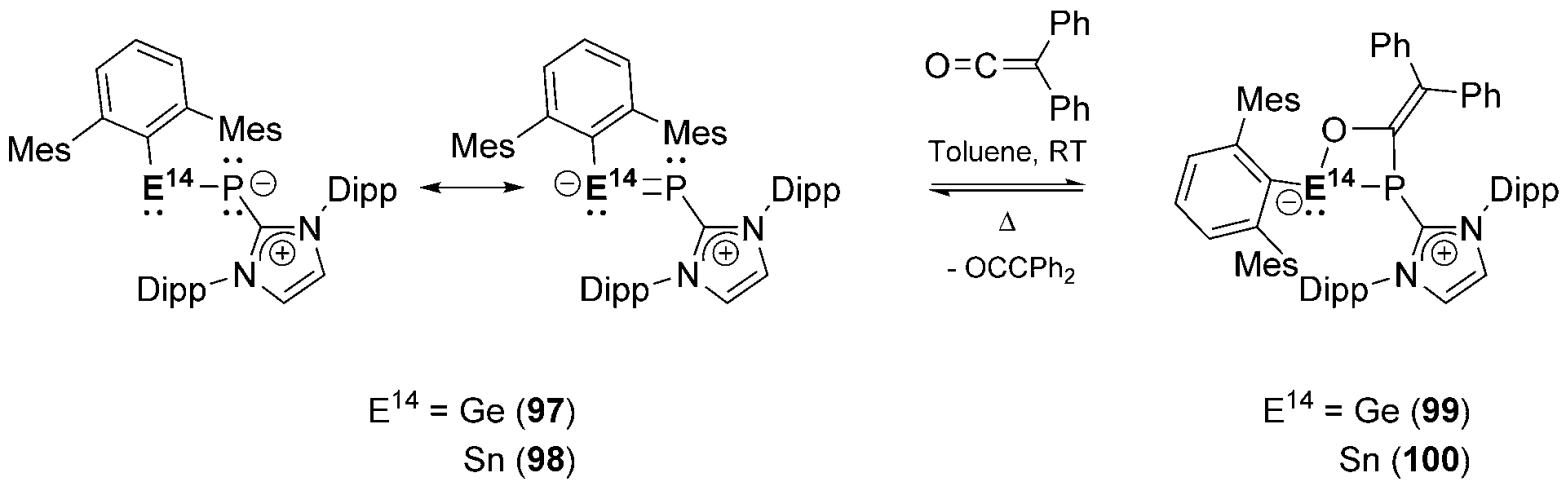
Heavier nitrile reversible reactivity with diphenylketene.

Heavier combinations of E^14^−E^15^ are also known, of which silaphosphines are the most common and have recently been reviewed.^[140]^ Examples of Ge and Sn‐phosphorous complexes are rare, but a recent example by Inoue and co‐workers showed that heavier nitrile analogues have interesting properties.^[141]^ Use of *N*‐heterocyclic phosphinidene (NHCPs) ligands allowed for the isolation of compounds **97** and **98** (Scheme 21). These compounds contain short E^14^−P bonds and were additionally shown through DFT calculations to contain some multiple bond character, due to the resulting resonance structures. Surprisingly, compounds **97** and **98** showed no reactivity towards small molecules (H_2_, CO and CO_2_), however interesting reversible reactivity towards diphenylketene was observed (Scheme 21). [2+2]‐cycloaddition occurs at room temperature, however, on heating **99** (80 °C) and **100** (100 °C) retro‐cycloaddition of the ketene was achieved.^[141]^

This reversibility prompted the examination of **97** and **98** in catalysis. Both compounds were found to be catalytically active in the hydroboration of aldehydes and ketones, with low loadings and fast reaction times at room temperature observed.^[141]^ This preliminary study highlights the potential for these heavier multiple bonds in catalysis. Particularly those of Ge and Sn wherein the stability of the lower oxidation state increases compared to the lighter congeners. Other recent achievements within E^14^−E^15^ multiple bonds have resulted in the first examples of a stibasilene^[142]^ (Si–Sb double bond) and an arsagermene^[143]^ (Ge‐As double bond). Albeit, no reactivity has been reported.

### E^14^–E^16^ multiple bonds

**Scheme 22:**
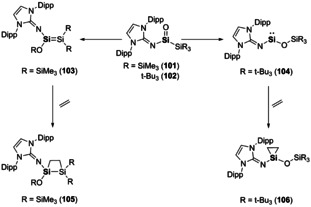
Ligand dependant transformations of silanones and subsequent reactivity with ethylene.

E^14^–E^16^ multiple bonds are probably the most widely studied, if you consider carbonyl containing compounds and efforts in the use of CO_2_ as a C1 feedstock for commodity chemicals.^[144]^ In contrast heavier carbonyls are rare due to the high polarity of the resulting E^14^−E^16^ bond. This, however, does have its advantages as poly(siloxanes) (R_2_SiO)_*n*_ have found widespread material use. It was not until the last decades that Kipping's dream^[145]^ was realised and now several examples of silanones (i.e. compounds containing a Si=O double bond) have been isolated.^[146]^ The high reactivity and instability of Si=O bonds was shown by Inoue and co‐workers, in which the first acyclic three‐coordinate silanones (**101** and **102**) were found to react with small molecules such as CO_2_ and methanol.^[147]^ The acyclic silanones (**101** and **102**) are indefinitely stable in the solid state but in solution they decompose readily (t_1/2_ for **101** is 7 h in C_6_D_6_). Interestingly, monitoring solutions of **101** and **102** revealed different migration pathways depending on the different substitution at the supporting silyl ligand (Scheme 22). In the case of SiMe_3_ substituents (**101**) a 1,3‐silyl migration was observed to form an intermediate disilene complex (**103**), analogous to keto‐enol isomerisation. However, use of a super silyl supporting ligand (*t*Bu_3_Si, Compound **102**) results in formation of a two‐coordinate *N,O*‐silylene (**104**). The differing reactivity of the two complexes was shown on reaction with ethylene (Scheme 22). The intermediate disilene (**103**) undergoes [2+2]‐cycloaddition to form a four‐membered ring (**105**) whereas the silylene (**104**) forms a three‐membered ring (**106**).^[147]^

**Scheme 23:**
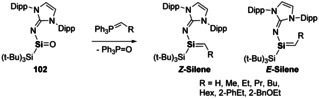
Sila‐Wittig reactivity of silanones, a new synthetic route to silenes.

Further reactivity of **102** has shown the potential of multiple bonds in synthesis. One of the versatile reactions in the organic chemist's toolkit is the Wittig reaction, wherein carbonyl compounds are used to prepare alkenes. As such, it was shown by Inoue and co‐workers that analogous reactivity is in fact possible with heavier carbonyls.^[148]^ This sila‐Wittig reactivity is shown to be possible with a range of non‐stabilised ylides (Scheme 23) resulting in high selectivity towards the *Z*‐silenes. This reactivity shows the similarities that can be found between carbon and silicon. Additionally, this provides new synthetic routes to silaalkenes.

**Scheme 24:**
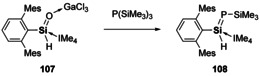
Silaphosphene synthesis from silaaldehydes. IMe_4_=1,3,4,5‐tetramethyl‐imidazol‐2‐ylidene.

Whilst silanones are somewhat established, examples of silaldehydes remain rare due to their reduced kinetic stabilisation. Lewis acid and base stabilisation methods enabled isolation of silaaldehydes, but single bond character was observed due to the push‐pull stabilisation.[^149^, ^150^, ^151^, ^152^, ^153^] Reports of the reactivity of such compounds are also scarce. However, the Lewis acid‐base stabilised silaaldehyde (**107**) revealed its carbonyl like reactivity (Scheme 24), on its reaction with phosphine which afforded the thermally stable silaphosphene (**108**).^[153]^

**Scheme 25:**
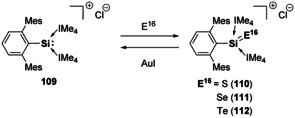
Si^II^/Si^IV^ formation via silicon chalcogen multiple bond formation and chalcogen abstraction.

Heavier E^14^–O multiple bonds, that is, Ge, Sn, and Pb, are known but are much rarer in comparison to the silicon analogues.^[146]^ The first example of a monomeric germanone was reported by Tamao and co‐workers. It was found to undergo a series of addition reactions highlighting the high nucleophilicity of the oxygen atom in comparison to ketones.^[154]^ Silanone analogues with heavier chalcogens, Si=E^16^ (E^16^=S, Se, Te) have been isolated, including the aldehyde analogues.[^155^, ^156^, ^157^, ^158^] The NHC‐stabilised cationic silyliumylidene **109** enabled access to a series of Si=E^16^ complexes.[^158^, ^159^, ^160^] The reaction of **109** with elemental S, Se or Te afforded the desired chalcogen complexes (compounds **110**–**112**, Scheme 25), with Si now in the +4 oxidation state.^[160]^ Interestingly, compound **109**, with Si in the +2 oxidation state, could be reformed on reaction with AuI, due to chalcogen transfer to the soft coinage metal. Additionally, chalcogen exchange reactions highlight preference for Si=S bond formation as the reaction of **111** and **112** with elemental sulphur results in the formation of compound **110**. No reactivity of **110** was observed with Se or Te.^[160]^

Descending group 14, examples of Ge−S, Se and Te multiple bonds exist.[^161^, ^162^, ^163^] Germanethiones and germaneselones were found to undergo a series of cycloadditions with unsaturated substrates, for example [2+2]‐cycloaddition with phenylisothiocyanate and [3+2]‐cycloaddition with mesitonitrile oxide.^[163]^

## Conclusions and Outlook

Main group multiple bonds have proven themselves to be a powerful tool in the modern main group chemist's toolkit. Whilst a large variety and combinations of E^13^/E^16^ multiple bonds now exist, the reactivity of these complexes has only really begun to emerge in the last decades. This has particularly been exemplified by the discovery that main group multiple bonds contain transition metal like properties and are therefore capable of facile activation of strong bonds such as dihydrogen. One of the key factors for the development of this chemistry has been the correct choice of supporting ligands. Not only has this enabled the isolation of the multiple bond, but also has a direct influence on the reactivity. For example, comparison of the recently isolated dialumenes (Al=Al double bond) moving from a silyl‐based ligand to an aryl ligand now enables dihydrogen activation. This is also observed with Braunschweig's diborynes (B≡B triple bond) with the difference in the ligands controlling the reactivity towards CO and H_2_.

Main group multiple bonds have also shown more than just small molecule reactivity. Their use in synthesis has also been highlighted enabling new routes to functionalised compounds. For example, the use of a boron‐oxygen double bond as an O‐transfer reagent, as well as silicon‐oxygen bonds in sila‐Wittig reactivity, the latter of which has enabled isolation of new silaethenes which also display novel reactivity.

Finally, examples of multiple bonds in catalysis have begun to emerge. Digermynes and dialumenes have shown the stability of these dinuclear systems is key to enabling turnover. It is anticipated that many more examples of catalytic application of main group multiple bonds will emerge over the next decade. With parallels being drawn to transition metals, catalytic cycles such as those highlighted in Scheme 1 will become more obtainable as further understanding of the intrinsic nature of metal‐metal bonds is realised.

## Conflict of interest

The authors declare no conflict of interest.

## Biographical Information

*Catherine Weetman obtained her MChem and Ph.D. at the University of Bath under the supervision of Prof. M. Hill in s‐block catalysis. Her first postdoctoral position was spent at the University of Edinburgh under Profs. P. Arnold and J. Love using d‐ and f‐block metals for bond activations and catalysis. Following this, she was awarded a postdoctoral fellowship to join Prof. S. Inoue at TU Munich*, *followed by a EuroTech Marie Curie Fellowship for Aluminium multiple bond chemistry which also included a six‐month research stay with Prof. M. Mazzanti at EPFL. In July 2020*, *Cath started her independent career at the University of Strathclyde as a Chancellor's Fellow*.

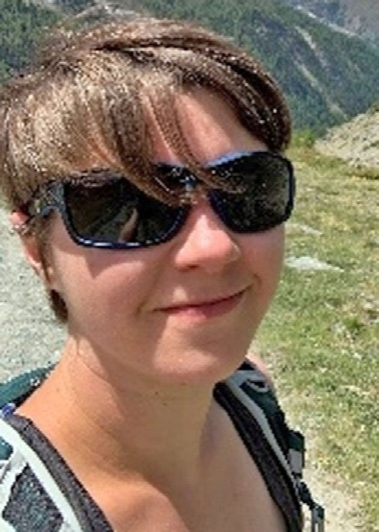

## References

[1] R. West , M. J. Fink , J. Michl , Science 1981, 214, 1343–1344. 17812259 10.1126/science.214.4527.1343

[2] M. Yoshifuji , I. Shima , N. Inamoto , K. Hirotsu , T. Higuchi , J. Am. Chem. Soc. 1981, 103, 4587–4589.

[3] A. G. Brook , F. Abdesaken , B. Gutekunst , G. Gutekunst , R. K. Kallury , J. Chem. Soc. Chem. Commun. 1981, 191–192.

[4] G. Becker , G. Gresser , W. Uhl , Z. Naturforsch. B 1981, 36, 16–19.

[5] P. P. Power , Chem. Rev. 1999, 99, 3463–3504. 11849028 10.1021/cr9408989

[6] R. C. Fischer , P. P. Power , Chem. Rev. 2010, 110, 3877–3923. 20672858 10.1021/cr100133q

[7] P. Bag , A. Porzelt , P. J. Altmann , S. Inoue , J. Am. Chem. Soc. 2017, 139, 14384–14387. 28898060 10.1021/jacs.7b08890

[8] H. Braunschweig , R. D. Dewhurst , K. Hammond , J. Mies , K. Radacki , A. Vargas , Science 2012, 336, 1420–1422. 22700924 10.1126/science.1221138

[9] F. S. Kipping , J. E. Sands , J. Chem. Soc. Trans. 1921, 119, 830–847.

[10] C. Dohmeier , C. Robl , M. Tacke , H. Schnockel , Angew. Chem. Int. Ed. Engl. 1991, 30, 564–565;

[11] A. Hofmann , T. Tröster , T. Kupfer , H. Braunschweig , Chem. Sci. 2019, 10, 3421–3428. 30996931 10.1039/c8sc05175e PMC6429597

[12] P. J. Davidson , M. F. Lappert , J. Chem. Soc. Chem. Commun. 1973, 317a.

[13] D. E. Goldberg , D. H. Harris , M. F. Lappert , K. M. Thomas , J. Chem. Soc. Chem. Commun. 1976, 261–262.

[14] P. P. Power , Nature 2010, 463, 171–177. 20075912 10.1038/nature08634

[15] C. Weetman , S. Inoue , ChemCatChem 2018, 10, 4213–4228.

[16] M. S. Hill , D. J. Liptrot , C. Weetman , Chem. Soc. Rev. 2016, 45, 972–988. 26797470 10.1039/c5cs00880h

[17] I. G. Powers , C. Uyeda , ACS Catal. 2017, 7, 936–958.

[18] S. T. Liddle , D. P. Mills , Dalton Trans. 2009, 5592–5605. 20449071 10.1039/b904318g

[19] T. Sugahara , J. D. Guo , T. Sasamori , S. Nagase , N. Tokitoh , Angew. Chem. Int. Ed. 2018, 57, 3499–3503; 10.1002/anie.201801222 29411488

[20] C. Weetman , P. Bag , T. Silvási , C. Jandl , S. Inoue , Angew. Chem. Int. Ed. 2019, 58, 10961–10968; 10.1002/anie.201905045 31112624

[21] J. Hicks , P. Vasko , J. M. Goicoechea , S. Aldridge , Nature 2018, 557, 92–95. 29662211 10.1038/s41586-018-0037-y

[22] J. Hicks , P. Vasko , J. M. Goicoechea , S. Aldridge , Angew. Chem. Int. Ed. 2020, 10.1002/anie.202007530; PMC7693242 32722863

[23] K. Hobson , C. J. Carmalt , C. Bakewell , Chem. Sci. 2020, 11, 6942–6956. 10.1039/d0sc02686g PMC8159300 34122993

[24] S. Fujimori , S. Inoue , Eur. J. Inorg. Chem. 2020, 3131–3142. 32999589 10.1002/ejic.202000479 PMC7507849

[25] R. Y. Kong , M. R. Crimmin , Dalton Trans. 2020, 10.1039/D0DT01564D.

[26] R. L. Melen , Science 2019, 363, 479–484. 30705183 10.1126/science.aau5105

[27] M. Zhong , S. Sinhababu , H. W. Roesky , Dalton Trans. 2020, 49, 1351–1364. 31942579 10.1039/c9dt04763h

[28] S. Yadav , S. Saha , S. S. Sen , ChemCatChem 2016, 8, 486–501.

[29] E. Rivard , P. P. Power , Inorg. Chem. 2007, 46, 10047–10064. 17975890 10.1021/ic700813h

[30] J. Moilanen , P. P. Power , H. M. Tuononen , Inorg. Chem. 2010, 49, 10992–11000. 21049909 10.1021/ic101487g

[31] D. J. Liptrot , P. P. Power , Nat. Rev. Chem. 2017, 1, 0004.

[32] G. Frenking , R. Tonner , S. Klein , N. Takagi , T. Shimizu , A. Krapp , K. K. Pandey , P. Parameswaran , Chem. Soc. Rev. 2014, 43, 5106–5139. 24916774 10.1039/c4cs00073k

[33] L. Zhao , M. Hermann , N. Holzmann , G. Frenking , Coord. Chem. Rev. 2017, 344, 163–204.

[34] V. Nesterov , D. Reiter , P. Bag , P. Frisch , R. Holzner , A. Porzelt , S. Inoue , Chem. Rev. 2018, 118, 9678–9842. 29969239 10.1021/acs.chemrev.8b00079

[35] Y. Wang , B. Quillian , P. Wei , C. S. Wannere , Y. Xie , R. B. King , H. F. Schaefer , P. V. R. Schleyer , G. H. Robinson , J. Am. Chem. Soc. 2007, 129, 12412–12413. 17887683 10.1021/ja075932i

[36] N. J. Hardman , R. L. Wright , A. D. Phillips , P. P. Power , Angew. Chem. Int. Ed. 2002, 41, 2842–2844; 10.1002/1521-3773(20020802)41:15<2842::AID-ANIE2842>3.0.CO;2-O 12203504

[37] R. J. Wright , A. D. Phillips , N. J. Hardman , P. P. Power , J. Am. Chem. Soc. 2002, 124, 8538–8539. 12121089 10.1021/ja026285s

[38] R. J. Wright , A. D. Phillips , S. Hino , P. P. Power , J. Am. Chem. Soc. 2005, 127, 4794–4799. 15796545 10.1021/ja0432259

[39] M. Arrowsmith , H. Braunschweig , T. E. Stennett , Angew. Chem. Int. Ed. 2017, 56, 96–115; 10.1002/anie.201610072 27860056

[40] P. Bag , C. Weetman , S. Inoue , Angew. Chem. Int. Ed. 2018, 57, 14394–14413; 10.1002/anie.201803900 29790227

[41] H. Braunschweig , T. Dellermann , R. D. Dewhurst , W. C. Ewing , K. Hammond , J. O. C. Jimenez-Halla , T. Kramer , I. Krummenacher , J. Mies , A. K. Phukan , A. Vargas , Nat. Chem. 2013, 5, 1025–1028. 24256866 10.1038/nchem.1778

[42] J. Böhnke , H. Braunschweig , T. Dellermann , W. C. Ewing , K. Hammond , J. O. C. Jimenez-Halla , T. Kramer , J. Mies , Angew. Chem. Int. Ed. 2015, 54, 13801–13805; 10.1002/anie.201506368 26427026

[43] M. Arrowsmith , J. Böhnke , H. Braunschweig , M. A. Celik , T. Dellermann , K. Hammond , Chem. Eur. J. 2016, 22, 17169–17172. 27685839 10.1002/chem.201604094

[44] A. Stoy , J. Böhnke , J. O. C. Jiménez-Halla , R. D. Dewhurst , T. Thiess , H. Braunschweig , Angew. Chem. Int. Ed. 2018, 57, 5947–5951; 10.1002/anie.201802117 29493885

[45] C. Weetman , A. Porzelt , P. Bag , F. Hanusch , S. Inoue , Chem. Sci. 2020, 11, 4817–4827. 10.1039/d0sc01561j PMC8159210 34122939

[46] Z. Zhu , X. Wang , Y. Peng , H. Lei , J. C. Fettinger , E. Rivard , P. P. Power , Angew. Chem. Int. Ed. 2009, 48, 2031–2034; 10.1002/anie.200805982 19180617

[47] C. A. Caputo , J.-D. Guo , S. Nagase , J. C. Fettinger , P. P. Power , J. Am. Chem. Soc. 2012, 134, 7155–7164. 22420622 10.1021/ja301247h

[48] C. A. Caputo , J. Koivistoinen , J. Moilanen , J. N. Boynton , H. M. Tuononen , P. P. Power , J. Am. Chem. Soc. 2013, 135, 1952–1960. 23343001 10.1021/ja3116789

[49] C. A. Caputo , P. P. Power , Organometallics 2013, 32, 2278–2286.

[50] R. J. Wright , M. Brynda , P. P. Power , Angew. Chem. Int. Ed. 2006, 45, 5953–5956; 10.1002/anie.200601925 16897794

[51] J. Su , X.-W. Li , R. C. Crittendon , G. H. Robinson , J. Am. Chem. Soc. 1997, 119, 5471–5472.

[52] A. Hofmann , M.-A. Légaré , L. Wüst , H. Braunschweig , Angew. Chem. Int. Ed. 2019, 58, 9776–9781; 10.1002/anie.201902655 30985966

[53] H. Braunschweig , I. Krummenacher , M.-A. Légaré , A. Matler , K. Radacki , Q. Ye , J. Am. Chem. Soc. 2017, 139, 1802–1805. 28103028 10.1021/jacs.6b13047

[54] M.-A. Légaré , G. Bélanger-Chabot , R. D. Dewhurst , E. Welz , I. Krummenacher , B. Engels , H. Braunschweig , Science 2018, 359, 896–900. 29472479 10.1126/science.aaq1684

[55] J. Zhao , B. Xu , W. Yu , X. Wang , Organometallics 2016, 35, 3272–3280.

[56] N. Nakata , A. Sekiguchi , J. Am. Chem. Soc. 2006, 128, 422–423. 16402824 10.1021/ja0570741

[57] D. Franz , T. Szilvási , A. Pöthig , S. Inoue , Chem. Eur. J. 2019, 25, 11036–11041. 31241215 10.1002/chem.201902877

[58] N. Nakata , A. Sekiguchi , Chem. Lett. 2007, 36, 662–663.

[59] E. M. Leitao , T. Jurca , I. Manners , Nat. Chem. 2013, 5, 817. 24056337 10.1038/nchem.1749

[60] M. A. Malik , M. Afzaal , P. O'Brien , Chem. Rev. 2010, 110, 4417–4446. 20481563 10.1021/cr900406f

[61] J. F. Li , X. F. Li , W. Huang , H. F. Hu , J. Y. Zhang , C. M. Cui , Chem. Eur. J. 2012, 18, 15263–15266. 23129126 10.1002/chem.201203298

[62] M. D. Anker , R. J. Schwamm , M. P. Coles , Chem. Commun. 2020, 56, 2288–2291. 10.1039/c9cc09214e 31984981

[63] A. Heilmann , J. Hicks , P. Vasko , J. M. Goicoechea , S. Aldridge , Angew. Chem. Int. Ed. 2020, 59, 4897–4901; 10.1002/anie.201916073 31999037

[64] R. J. Wright , M. Brynda , J. C. Fettinger , A. R. Betzer , P. P. Power , J. Am. Chem. Soc. 2006, 128, 12498–12509. 16984201 10.1021/ja063072k

[65] N. J. Hardman , C. Cui , H. W. Roesky , W. H. Fink , P. P. Power , Angew. Chem. Int. Ed. 2001, 40, 2172–2174; 10.1002/1521-3773(20010601)40:11<2172::AID-ANIE2172>3.0.CO;2-Y 29712210

[66] R. J. Wright , A. D. Phillips , T. L. Allen , W. H. Fink , P. P. Power , J. Am. Chem. Soc. 2003, 125, 1694–1695. 12580583 10.1021/ja029422u

[67] M. D. Anker , M. Lein , M. P. Coles , Chem. Sci. 2019, 10, 1212–1218. 30774921 10.1039/c8sc04078h PMC6349055

[68] E. Rivard , W. A. Merrill , J. C. Fettinger , P. P. Power , Chem. Commun. 2006, 3800–3802. 10.1039/b609748k 16969462

[69] E. Rivard , W. A. Merrill , J. C. Fettinger , R. Wolf , G. H. Spikes , P. P. Power , Inorg. Chem. 2007, 46, 2971–2978. 17338516 10.1021/ic062076n

[70] G. Linti , H. Nöth , K. Polborn , R. T. Paine , Angew. Chem. Int. Ed. Engl. 1990, 29, 682–684;

[71] A. N. Price , M. J. Cowley , Chem. Eur. J. 2016, 22, 6248–6252. 26918876 10.1002/chem.201600836

[72] A. N. Price , G. S. Nichol , M. J. Cowley , Angew. Chem. Int. Ed. 2017, 56, 9953–9957; 10.1002/anie.201705050 28643472

[73] C. M. E. Graham , C. R. P. Millet , A. N. Price , J. Valjus , M. J. Cowley , H. M. Tuononen , P. J. Ragogna , Chem. Eur. J. 2018, 24, 672–680. 29119625 10.1002/chem.201704337

[74] J. R. Davis , Aluminum and Aluminum Alloys, ASM International, Almere, 1993.

[75] D. Franz , S. Inoue , Dalton Trans. 2016, 45, 9385–9397. 27216700 10.1039/c6dt01413e

[76] H. Braunschweig , K. Radacki , A. Schneider , Science 2010, 328, 345–347. 20395506 10.1126/science.1186028

[77] Y. K. Loh , K. Porteous , M. Á. Fuentes , D. C. H. Do , J. Hicks , S. Aldridge , J. Am. Chem. Soc. 2019, 141, 8073–8077. 31046264 10.1021/jacs.9b03600

[78] F. Dielmann , O. Back , M. Henry-Ellinger , P. Jerabek , G. Frenking , G. Bertrand , Science 2012, 337, 1526–1528. 22997335 10.1126/science.1226022

[79] M. D. Anker , M. P. Coles , Angew. Chem. Int. Ed. 2019, 58, 18261–18265; 10.1002/anie.201911550 31568609

[80] J. Hicks , A. Heilmann , P. Vasko , J. Goicoechea , S. Aldridge , Angew. Chem. Int. Ed. 2019, 58, 17265–17268; 10.1002/anie.201910509 31550066

[81] M. D. Anker , C. L. McMullin , N. A. Rajabi , M. P. Coles , Angew. Chem. Int. Ed. 2020, 59, 12806–12810. 10.1002/anie.202005301 32378311

[82] M. D. Anker , M. P. Coles , Angew. Chem. Int. Ed. 2019, 58, 13452—134550; 10.1002/anie.201907884 31295385

[83] D. Franz , T. Szilvasi , E. Irran , S. Inoue , Nat. Commun. 2015, 6, 10037–10042. 26612781 10.1038/ncomms10037 PMC4674676

[84] M. C. Kuchta , G. Parkin , J. Am. Chem. Soc. 1995, 117, 12651–12652.

[85] M. C. Kuchta , J. B. Bonanno , G. Parkin , J. Am. Chem. Soc. 1996, 118, 10914–10915.

[86] M. C. Kuchta , G. Parkin , Inorg. Chem. 1997, 36, 2492–2493.

[87] M. C. Kuchta , G. Parkin , J. Chem. Soc. Dalton Trans. 1998, 2279–2280.

[88] J. Clayden , N. Greeves , S. Warren , Organic Chemistry , 2nd ed., Oxford University Press, Oxford, 2012.

[89] C. Präsang , D. Scheschkewitz , Chem. Soc. Rev. 2016, 45, 900–921. 26503807 10.1039/c5cs00720h

[90] A. Rammo , D. Scheschkewitz , Chem. Eur. J. 2018, 24, 6866–6885. 29193341 10.1002/chem.201704090

[91] M. Kira , T. Iwamoto in Advances in Organometallic Chemistry, Vol. 54 (Eds.: R. West , A. F. Hill ), Academic Press, 2006, pp. 73–148.

[92] P. P. Power , J. Chem. Soc. Dalton Trans. 1998, 2939–2951.

[93] A. Sekiguchi , R. Kinjo , M. Ichinohe , Science 2004, 305, 1755–1757. 15375262 10.1126/science.1102209

[94] M. Stender , A. D. Phillips , R. J. Wright , P. P. Power , Angew. Chem. Int. Ed. 2002, 41, 1785–1787; 10.1002/1521-3773(20020517)41:10<1785::aid-anie1785>3.0.co;2-6 19750717

[95] A. D. Phillips , R. J. Wright , M. M. Olmstead , P. P. Power , J. Am. Chem. Soc. 2002, 124, 5930–5931. 12022812 10.1021/ja0257164

[96] L. Pu , B. Twamley , P. P. Power , J. Am. Chem. Soc. 2000, 122, 3524–3525.

[97] J.-D. Guo , T. Sasamori , Chem. Asian J. 2018, 13, 3800–3817. 30320958 10.1002/asia.201801329

[98] G. H. Spikes , J. C. Fettinger , P. P. Power , J. Am. Chem. Soc. 2005, 127, 12232–12233. 16131195 10.1021/ja053247a

[99] D. Wendel , T. Szilvási , C. Jandl , S. Inoue , B. Rieger , J. Am. Chem. Soc. 2017, 139, 9156–9159. 28640616 10.1021/jacs.7b05335

[100] D. Wendel , T. Szilvási , D. Henschel , P. J. Altmann , C. Jandl , S. Inoue , B. Rieger , Angew. Chem. Int. Ed. 2018, 57, 14575–14579; 10.1002/anie.201804472 29920891

[101] T. Kosai , T. Iwamoto , J. Am. Chem. Soc. 2017, 139, 18146–18149. 29192775 10.1021/jacs.7b09989

[102] T. Kosai , T. Iwamoto , Chem. Eur. J. 2018, 24, 7774–7780. 29604135 10.1002/chem.201801286

[103] D. Reiter , R. Holzner , A. Porzelt , P. J. Altmann , P. Frisch , S. Inoue , J. Am. Chem. Soc. 2019, 141, 13536–13546. 31352777 10.1021/jacs.9b05318

[104] M. W. Stanford , J. I. Schweizer , M. Menche , G. S. Nichol , M. C. Holthausen , M. J. Cowley , Angew. Chem. Int. Ed. 2019, 58, 1329–1333; 10.1002/anie.201810056 30461143

[105] H. W. Roesky , J. Organomet. Chem. 2013, 730, 57–62.

[106] B. Blom , D. Gallego , M. Driess , Inorg. Chem. Front. 2014, 1, 134–148.

[107] N. Muthukumaran , K. Velappan , K. Gour , G. Prabusankar , Coord. Chem. Rev. 2018, 377, 1–43.

[108] T. Sasamori , T. Sugahara , T. Agou , J.-D. Guo , S. Nagase , R. Streubel , N. Tokitoh , Organometallics 2015, 34, 2106–2109.

[109] T. Sugahara , J.-D. Guo , T. Sasamori , Y. Karatsu , Y. Furukawa , A. E. Ferao , S. Nagase , N. Tokitoh , Bull. Chem. Soc. Jpn. 2016, 89, 1375–1384.

[110] P. P. Power , Chem. Commun. 2003, 2091–2101. 10.1039/b212224c 13678155

[111] T. Y. Lai , L. Tao , R. D. Britt , P. P. Power , J. Am. Chem. Soc. 2019, 141, 12527–12530. 31345027 10.1021/jacs.9b06845

[112] S. Wang , T. J. Sherbow , L. A. Berben , P. P. Power , J. Am. Chem. Soc. 2018, 140, 590–593. 29272120 10.1021/jacs.7b11798

[113] J. D. Queen , M. Bursch , J. Seibert , L. R. Maurer , B. D. Ellis , J. C. Fettinger , S. Grimme , P. P. Power , J. Am. Chem. Soc. 2019, 141, 14370–14383. 31390863 10.1021/jacs.9b07072

[114] V. Y. Lee , A. Sekiguchi , Organometallics 2004, 23, 2822–2834.

[115] A. Sekiguchi , V. Y. Lee , Chem. Rev. 2003, 103, 1429–1448. 12683787 10.1021/cr0100300

[116] H. Ottosson , A. M. Eklöf , Coord. Chem. Rev. 2008, 252, 1287–1314.

[117] L. E. Gusel′nikov , Coord. Chem. Rev. 2003, 244, 149–240.

[118] K. K. Milnes , L. C. Pavelka , K. M. Baines , Chem. Soc. Rev. 2016, 45, 1019–1035. 26335610 10.1039/c5cs00522a

[119] W.-P. Leung , Y.-C. Chan , C.-W. So , Organometallics 2015, 34, 2067–2085.

[120] C. Bibal , S. Mazières , H. Gornitzka , C. Couret , Angew. Chem. Int. Ed. 2001, 40, 952–954; 11241657

[121] W. Setaka , K. Hirai , H. Tomioka , K. Sakamoto , M. Kira , J. Am. Chem. Soc. 2004, 126, 2696–2697. 14995175 10.1021/ja0389974

[122] D. Gau , T. Kato , N. Saffon-Merceron , A. De Cózar , F. P. Cossío , A. Baceiredo , Angew. Chem. Int. Ed. 2010, 49, 6585–6588; 10.1002/anie.201003616 20677192

[123] T. L. Windus , M. S. Gordon , J. Am. Chem. Soc. 1992, 114, 9559–9568.

[124] A. Sekiguchi , R. Izumi , V. Y. Lee , M. Ichinohe , J. Am. Chem. Soc. 2002, 124, 14822–14823. 12475304 10.1021/ja021077l

[125] A. Schäfer , W. Saak , M. Weidenbruch , Organometallics 2003, 22, 215–217.

[126] G. He , O. Shynkaruk , M. W. Lui , E. Rivard , Chem. Rev. 2014, 114, 7815–7880. 24605824 10.1021/cr400547x

[127] T. Iwamoto , H. Masuda , C. Kabuto , M. Kira , Organometallics 2005, 24, 197–199.

[128] J. Escudié , H. Ranaivonjatovo , Organometallics 2007, 26, 1542–1559.

[129] G. Frenking , M. Hermann , D. M. Andrada , N. Holzmann , Chem. Soc. Rev. 2016, 45, 1129–1144. 26815221 10.1039/c5cs00815h

[130] P. Le Floch , Coord. Chem. Rev. 2006, 250, 627–681.

[131] F. Mathey , Angew. Chem. Int. Ed. 2003, 42, 1578–1604; 10.1002/anie.200200557 12698454

[132] M. Denk , R. K. Hayashi , R. West , J. Am. Chem. Soc. 1994, 116, 10813–10814.

[133] M. Hesse , U. Klingebiel , Angew. Chem. Int. Ed. Engl. 1986, 25, 649–650;

[134] L. B. Kong , C. M. Cui , Organometallics 2010, 29, 5738–5740.

[135] P. P. Samuel , R. Azhakar , R. S. Ghadwal , S. S. Sen , H. W. Roesky , M. Granitzka , J. Matussek , R. Herbst-Irmer , D. Stalke , Inorg. Chem. 2012, 51, 11049–11054. 23036040 10.1021/ic301543y

[136] K. Yuvaraj , C. Jones , Dalton Trans. 2019, 48, 11961–11965. 31318370 10.1039/c9dt02657f

[137] J. Barrau , J. Escudie , J. Satge , Chem. Rev. 1990, 90, 283–319.

[138] A. Rit , R. Tirfoin , S. Aldridge , Angew. Chem. Int. Ed. 2016, 55, 378–382; 10.1002/anie.201508940 26545498

[139] M. J. Evans , M. D. Anker , A. Mouchfiq , M. Lein , J. R. Fulton , Chem. Eur. J. 2020, 26, 2606–2609. 31863493 10.1002/chem.201905693

[140] V. Nesterov , N. C. Breit , S. Inoue , Chem. Eur. J. 2017, 23, 12014–12039. 28379639 10.1002/chem.201700829

[141] V. Nesterov , R. Baierl , F. Hanusch , A. E. Ferao , S. Inoue , J. Am. Chem. Soc. 2019, 141, 14576–14580. 31476856 10.1021/jacs.9b08741

[142] V. Y. Lee , S. Aoki , M. Kawai , T. Meguro , A. Sekiguchi , J. Am. Chem. Soc. 2014, 136, 6243–6246. 24742269 10.1021/ja5026084

[143] V. Y. Lee , M. Kawai , O. A. Gapurenko , V. I. Minkin , H. Gornitzka , A. Sekiguchi , Chem. Commun. 2018, 54, 10947–10949. 10.1039/c8cc05630g 30105326

[144] S. D. Allen , C. M. Byrne , G. W. Coates in Feedstocks for the Future, Vol. 921, American Chemical Society, 2006, pp. 116–129.

[145] F. S. Kipping , L. L. Lloyd , J. Chem. Soc. Trans. 1901, 79, 449–459.

[146] Y. Xiong , S. Yao , M. Driess , Angew. Chem. Int. Ed. 2013, 52, 4302–4311; 10.1002/anie.201209766 23450830

[147] D. Wendel , D. Reiter , A. Porzelt , P. J. Altmann , S. Inoue , B. Rieger , J. Am. Chem. Soc. 2017, 139, 17193–17198. 29098861 10.1021/jacs.7b10634

[148] D. Reiter , P. Frisch , T. Szilvási , S. Inoue , J. Am. Chem. Soc. 2019, 141, 16991–16996. 31560854 10.1021/jacs.9b09379

[149] S. Yao , M. Brym , C. van Wüllen , M. Driess , Angew. Chem. Int. Ed. 2007, 46, 4159–4162; 10.1002/anie.200700398 17436257

[150] R. S. Ghadwal , R. Azhakar , H. W. Roesky , K. Pröpper , B. Dittrich , C. Goedecke , G. Frenking , Chem. Commun. 2012, 48, 8186–8188. 10.1039/c2cc32887a 22786582

[151] T. Fukuda , H. Hashimoto , S. Sakaki , H. Tobita , Angew. Chem. Int. Ed. 2016, 55, 188–192; 10.1002/anie.201507956 26768821

[152] D. C. H. Do , A. V. Protchenko , M. Ángeles Fuentes , J. Hicks , E. L. Kolychev , P. Vasko , S. Aldridge , Angew. Chem. Int. Ed. 2018, 57, 13907–13911; 10.1002/anie.201807543 30168242

[153] D. Sarkar , V. Nesterov , T. Szilvási , P. J. Altmann , S. Inoue , Chem. Eur. J. 2019, 25, 1198–1202. 30444958 10.1002/chem.201805604

[154] L. Li , T. Fukawa , T. Matsuo , D. Hashizume , H. Fueno , K. Tanaka , K. Tamao , Nat. Chem. 2012, 4, 361–365. 22522255 10.1038/nchem.1305

[155] A. Burchert , R. Müller , S. Yao , C. Schattenberg , Y. Xiong , M. Kaupp , M. Driess , Angew. Chem. Int. Ed. 2017, 56, 6298–6301; 10.1002/anie.201700530 28394041

[156] D. Lutters , A. Merk , M. Schmidtmann , T. Müller , Inorg. Chem. 2016, 55, 9026–9032. 27513007 10.1021/acs.inorgchem.6b01510

[157] F. M. Mück , D. Kloß , J. A. Baus , C. Burschka , R. Bertermann , J. Poater , C. Fonseca Guerra , F. M. Bickelhaupt , R. Tacke , Chem. Eur. J. 2015, 21, 14011–14021. 26284318 10.1002/chem.201501789

[158] A. Porzelt , I. J. Schweizer , R. Baierl , J. P. Altmann , C. M. Holthausen , S. Inoue , Inorganics 2018, 6, 54–68.

[159] S. U. Ahmad , T. Szilvási , E. Irran , S. Inoue , J. Am. Chem. Soc. 2015, 137, 5828–5836. 25871835 10.1021/jacs.5b01853

[160] D. Sarkar , D. Wendel , S. U. Ahmad , T. Szilvási , A. Pöthig , S. Inoue , Dalton Trans. 2017, 46, 16014–16018. 29120473 10.1039/c7dt03998k

[161] Y. Xiong , S. Yao , M. Karni , A. Kostenko , A. Burchert , Y. Apeloig , M. Driess , Chem. Sci. 2016, 7, 5462–5469. 30034685 10.1039/c6sc01839d PMC6021755

[162] N. Tokitoh , T. Matsumoto , K. Manmaru , R. Okazaki , J. Am. Chem. Soc. 1993, 115, 8855–8856.

[163] T. Matsumoto , N. Tokitoh , R. Okazaki , J. Am. Chem. Soc. 1999, 121, 8811–8824.

